# Rapid purification of giant lipid vesicles by microfiltration

**DOI:** 10.1371/journal.pone.0192975

**Published:** 2018-02-16

**Authors:** Dimitri Fayolle, Michele Fiore, Pasquale Stano, Peter Strazewski

**Affiliations:** 1 Institut de Chimie et Biochimie Moléculaires et Supramoléculaires, Université de Lyon, Claude Bernard Lyon 1, Villeurbanne Cedex, France; 2 Department of Sciences, Roma Tre University, Rome, Italy; Nagoya University, JAPAN

## Abstract

Giant lipid vesicles (GVs) are emerging models for investigating the properties and reactivity of cell-like microcompartments, providing useful information about plausible protocellular structures in primitive times, as well as for the modern synthetic biology goal of constructing the first artificial cell from its reconstituted and partly modified components. Here we explore a novel methodology of GV purification by microfiltration under reduced pressure, operated by a simple apparatus. The method has been characterized in terms of flow rate, amount of lipid loss, quality of recovered GVs, and size distribution. A case study is reported to show the practicability of GV microfiltration. A clickable fluorescent probe was encapsulated inside GVs; more than 99.9% of the non-entrapped probe was easily and rapidly removed by multiple microfiltrations. This novel methodology is briefly discussed as a future tool for selection experiments on GV populations.

## 1. Introduction

Lipid vesicles (or liposomes) are supramolecular cell-like structures originating from the self-assembly of lipids in aqueous solutions. The lipid bilayer constitutes a continuous and generally spherical semi-permeable membrane enclosing an aqueous compartment. Small and large molecules can be incorporated either in the aqueous lumen or in the vesicle membrane, allowing the construction of simplified cellular models.

In the recent years, an ever-increasing number of reports have focused on the so-called giant vesicles (GVs), which, thanks to their very large size (typically 1–50 μm diameter), can be directly observed by conventional microscopy. Several studies have been published on the use of GVs as membrane models [1–3] and as cellular models, either in origin-of-life protocell research [4–9], or for artificial cell-like systems in the context of synthetic biology [10–13].

GV preparation requires special methods [14], which differ from the classical procedures used in the case of sub-micrometer (conventional) vesicles. Three methods are widely used to prepare GVs, namely, natural swelling [15,16], electroswelling [17] and emulsion droplet transfer [11,18]. These methods allow for the encapsulation of solutes in the GV’s lumen, such as hydrophilic fluorescent markers, enzymes, and nucleic acids. This is easily done by including the solutes of interest in the aqueous solutions used for preparing the GVs.

Irrespective of the preparation method, the crude GV suspension will contain non-entrapped solute molecules. For many applications, the GVs need to be purified from non-entrapped solutes that might interfere in successive measurements or reactions.

GVs are typically purified by dialysis or by differential centrifugation. Dialysis is time-consuming (several hours) and requires a large volume of external solution. In some applications, dialysis can be disadvantageous because the external solution can be expensive or difficult to prepare. Prolonged times also might be a drawback, especially if GVs contain sensitive molecules. Differential centrifugation is more rapid than dialysis (10–15 minutes), but it requires a density difference between the inner and the outer solutions, which is generally achieved by incorporating relatively high concentrations of sucrose inside GVs and glucose in the outer solution. The centrifuged GVs, when collected, might include part of their surrounding solution, impairing the purification, and more centrifugation rounds are thus required; this increases the overall purification time and reduces the number of recovered GVs.

As part of our ongoing research on the production, manipulation and usage of GVs, we were interested in exploring alternative purification methods. Here we report the microfiltration of GVs by means of a thin nylon membrane with narrow pores (0.2 μm). When coupled to a dilution of the GV sample, this procedure allows for a very effective removal of non-entrapped solutes (> 99.9%), is faster than dialysis or centrifugation, does not require a density difference, and can be used for volume samples of for example 10 mL being diminished to ca. 500 μL; below this minimal volume it is difficult to finely control the filtration end point and/or recover the GV suspension from the filter. This method is very practical and useful, even if GV recovery is not very high, depending on conditions, between 10% and 60% after each filtration step.

Interestingly, since the filter acts as a physical support for GVs, our approach can also be used for *in situ* repetitive GV manipulations, i.e., addition of first reactants to GVs over the filter, wash, filter, addition of second reactants, and so on. In this way, it becomes possible to investigate vesicle transformations, intra-vesicle or membrane-localized reactions, and design vesicle competition or selection experiments.

## 2. Results

**Fig 1:**
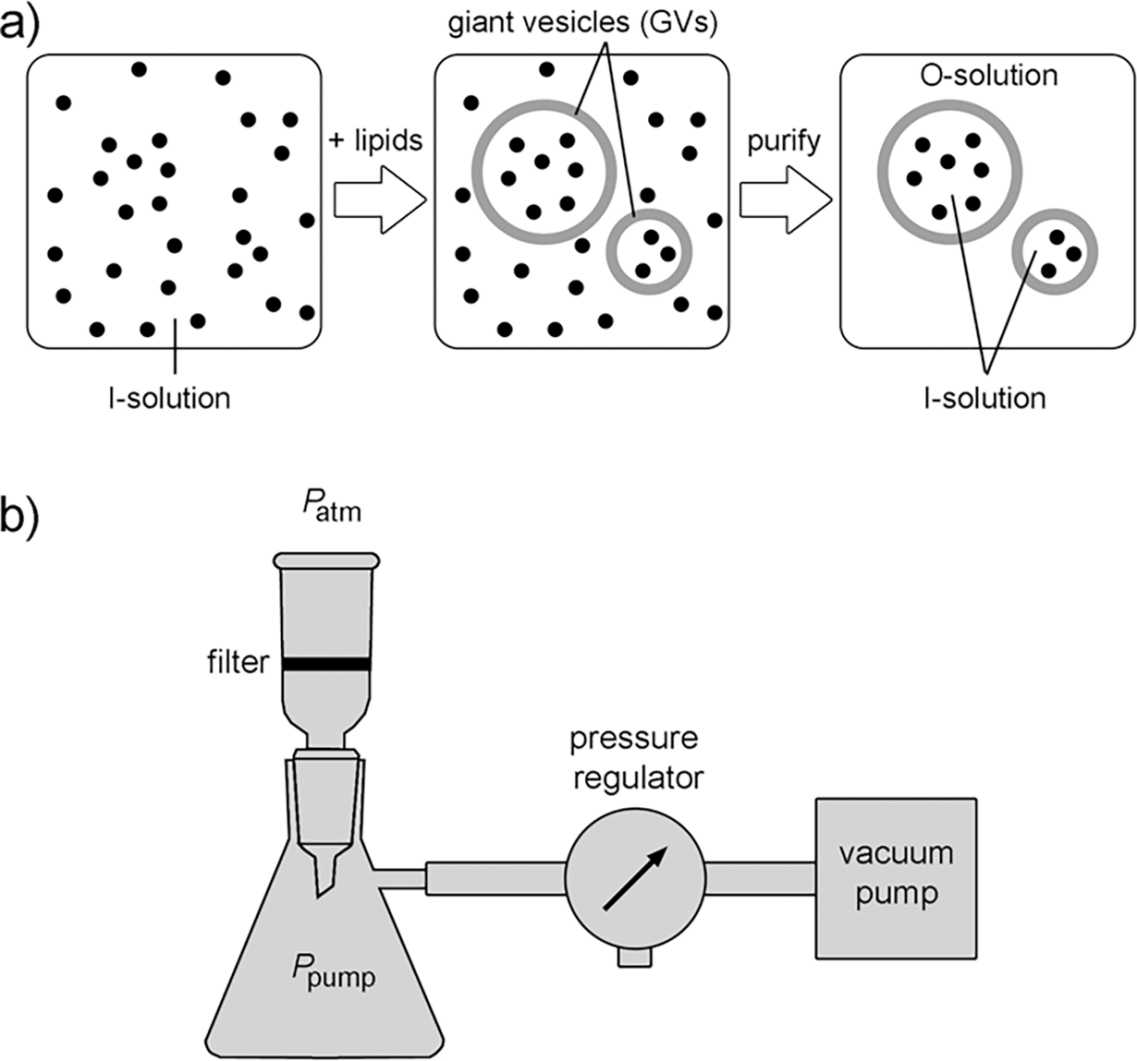
Purification of giant vesicles by microfiltration. (a) Giant lipid vesicles (GVs) are formed starting from an I-solution, that contains the solute of interest (indicated by black dots), by lipid hydration. Most of the solutes are not entrapped, and are found in the external solution; their removal is the goal of GV purification. Ideally, an isotonic O-solution should completely replace the original external solution. (b) Schematic drawing of microfiltration apparatus. Nylon filters with 0.2 μm pores have been used in this study. Note that Δ*P* = *P*_atm_−*P*_pump_ ≈ 1000 mbar–*P*_pump_.

The encapsulation inside vesicles of one or more types of water-soluble solutes with negligible transmembrane permeability is a very common situation met in most studies. GVs are prepared by allowing lipid self-assembly in a solution of the solutes of interest, *i*.*e*., those that need to be encapsulated in the vesicle lumen. Immediately after vesicle preparation most of the solute molecules of the so-called ‘I-solution’ (inside solution) are statistically found outside the vesicles, since the sum of all individual lumen volumes is generally only a few percent of the total volume (Fig 1A). To remove the non-entrapped molecules from the crude GV suspension, the external aqueous phase must be replaced by an ‘O-solution’ (outside solution) being usually isotonic with the I-solution, except when an osmotic stress is intentionally applied.

In this study, we have used GVs prepared by the natural swelling method. This method is well known and widely used [14], although it gives GVs with a broad size distribution, and, depending on the conditions, vesicles with more than one membrane (*i*.*e*., giant unilamellar vesicles, GUVs, and giant oligolamellar vesicles–indeed a detailed study of GVs lamellarity prepared in similar conditions has been reported [19]). DOPC was the principal membrane component. Occasionally DOPC was mixed with 30 mol% DOPA, and other lipid mixtures have also been tested. I- and O-solutions consisted in low-concentration buffers, such as 5 mM Na-bicine (pH 8.5) or 25 mM Tris-HCl (pH 7.5). The good stability of these GVs allowed easy manipulation and storage.

### 2.1 Apparatus and procedure

The removal of the external solution from a GV suspension by microfiltration requires a selective passage of solutes through the pores of the filter while GVs are retained in the ‘retentate’. To this end, nylon filters of a microscopic sponge-like structure with 0.2 μm average pore size have been used (see Materials and Methods). GV microfiltration was performed on a simple apparatus that can be easily assembled in most of laboratories (Fig 1B). It consists of a small filter holder, which is funnel-shaped, connected to a vacuum Erlenmeyer flask. A pump with a pressure regulator is connected to the flask, to generate across the filter a pressure difference (Δ*P* = *P*_atm_−*P*_pump_), which drives the microfiltration.

Unlike centrifugation, GV filtration does not require any density difference between the lumen and outside solution, so in principle no GV pre-treatment is needed. It is however convenient to dilute the crude GVs with O-solution, in order to improve the filtration efficiency and avoid filter occlusion (see below).

When a volume *V* of the GV suspension is diluted with a volume *V*_*O*_ of O-solution the concentration of non-entrapped solutes decreases by a factor *b* (Eq 1).

$$b=\frac{V+V_{O}}{V}$$ (Eq 1)

Although this operation does not change the intra-vesicle solute concentration, it reduces the GV concentration. When a diluted GV suspension *V* + *V*_*O*_ is filtered, the GVs are retained by the filter and become concentrated in the retentate by a factor *β* (Eq 2),

$$\beta =\frac{V+V_{O}}{V_{ret}}$$ (Eq 2)

where *V*_ret_ is the volume of the retentate. During filtration, small external solutes maintain their concentration, but are reduced in number. It follows that combining a preliminary GV dilution with a subsequent GV microfiltration is an optimal and rapid route to effectively purify GVs. For example, if GVs are first diluted 20 times (*b* = 20) with the O-solution, and this diluted sample is next filtered down to a 10-fold volume reduction (*β* = 10), one ideally gets in the retentate a 20-fold reduction of external solute concentration, and a mere 2-fold reduction of GV concentration. As it will be shown below, however, the GV recovery in the retentate is lower than the expected because of vesicle adsorption/rupture on the filter.

### 2.2 Factors affecting microfiltration efficiency

The efficiency of GVs microfiltration depends on several variables. Sample-dependent factors can be the type of lipids and their concentration, the vesicle size, the viscosity of the solution, the type of solutes that need to be drained away. Instrumental factors are the filter material, the filter surface and its thickness, the filter pore size, and the applied pressure difference Δ*P*. In this work we have investigated two key variables, namely, the lipid concentration and the applied pressure difference. Occasionally, we have changed the GVs lipid composition.

The effectiveness of the method was evaluated using three independently measurable parameters, the filtration time (and the related flow rate), the percent amount of GVs recovered in the retentate (the ‘recovery rate’, measured from lipid concentration) and the success of external solute removal, the latter being measured by the ratio of the solute concentration inside (*C*_in_) over the solute concentration outside GVs (*C*_out_) in the retentate sample, termed signal-to-noise ratio (SNR, *i*.*e*., *C*_in_/*C*_out_).

#### 2.2.1 Lipid concentration

The lipid concentration is a critical parameter of GV microfiltration. Preliminary experiments showed that the microfiltration of a GV suspension (10 mL, 300 μM) could not proceed to completion (e.g., to 10-fold reduction of the initial volume) because the filter became clogged, irrespectively of the applied Δ*P*. These initial observations suggest that the filter clogging is possibly due to GV debris, derived from the lipid adhesion to the filter material and facilitated by the applied suction force.

**Fig 2:**
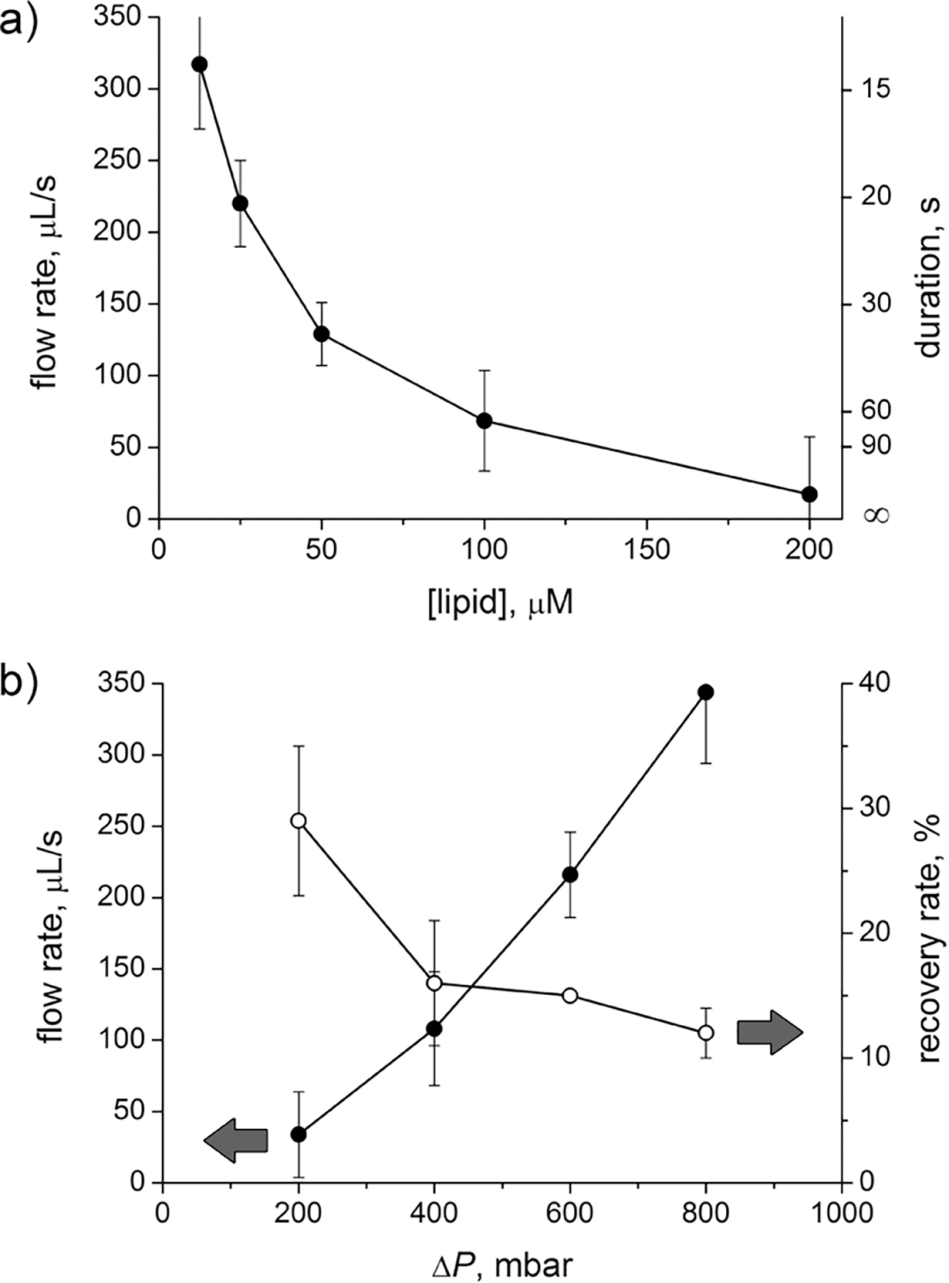
Parameters impacting on microfiltration. (a) Effect of lipid concentration on the flow rate and filtration duration, as measured for DOPC GVs (in 5 mM Na-bicine, pH 8.5). Filtration data refer to the reduction of an initial volume of 5 mL to 0.5 mL (*β* = 10); *P*_pump_ = 600 mbar, Δ*P* = 400 mbar. (b) Effect of pressure difference, Δ*P*, on the flow rate and recovery rate measured for DOPC GVs (in 25 mM Tris-HCl, pH 7.5). Data refer to the volume reduction from 5 mL to 0.5 mL (*β* = 10) of 50 μM DOPC GVs (250 nmoles applied over the filter).

Clearly, an ideal filtration process would require minimal GV-filter interaction and minimal Δ*P*, in order to preserve GV’s integrity. Such a setup would result, however, in very long filtration times. Systematic experiments were carried out, in order to find the best compromise between GV concentration and filtration efficiency in terms of short filtration time and no clogging. GV samples of different lipid concentrations were filtered at Δ*P* = 400 mbar in order to obtain a 10-fold volume reduction (from 5 to 0.5 mL). The dependence of average flow rates and filtration times on lipid concentration is reported in Fig 2A. For example, the filtration of 50 μM DOPC GVs takes ca. 35 s, whereas more concentrated vesicle suspensions require disproportionately longer times, e.g., the filtration of a 4-fold higher GV concentration takes a 7-fold longer filtration time (ca. 4 minutes for 200 μM DOPC GVs). Samples with higher than 200 μM lipid concentrations easily lead to filter clogging. Additional experiments indicate that flow rates and the moment of filter clogging can depend on the type of lipid, especially when oleate/oleic acid vesicles are abundant. Flow rates measurements showed in our hands a certain variance (shown as error bar in Fig 2), which is around 15% for most conditions, but increases when the filtration become too slow.

These evidences suggest that it is convenient to filtrate GVs while keeping the lipid concentration low. Next, we explored whether applying a higher Δ*P* could increase the flow rate.

#### 2.2.2 Influence of the pressure on the flow rate and the lipid loss

Under the same experimental conditions as specified above, microfiltration was carried out by applying different Δ*P*; the resulting flow rates are shown in Fig 2B. Flow rates increase by increasing Δ*P*, and filtration can be completed in shorter times. Except for very small Δ*P*, less than one minute is generally required for completing a GV filtration.

In order to quantify the amount of recovered GVs, the lipid concentration of the retentate was measured by the colorimetric Stewart assay [20]. In the mentioned experimental settings, recovery rates are always less than 30% (Fig 2B), meaning that most of GVs are lost by interaction with the filter. GVs could be either ‘extruded’ through the filter, passing in the filtrate as small vesicles, or broken so that the resulting debris gets adsorbed or stacked on the filter surface. When GV membranes were marked pink, by co-hydration of DOPC containing 0.1% mol DOPE-Rh, the filter was stained pink, suggesting that most lipids stay attached to the filter. The lipid concentration in the filtrate was also assayed, resulting in concentrations below the detection limit (< 6 μM). These observations suggest that lipid loss is largely due to lipid-filter interaction bringing about lipid retention on the filter surface.

The best compromise for most microfiltration operations can be achieved by applying a moderate vacuum, i.e., Δ*P =* 400–600 mbar.

#### 2.2.3 Extent of solute removal and the strategy of successive filtrations

The extent of solute removal obtained by GV microfiltration was then tested. As mentioned above, our protocol involves a preliminary dilution step with the O-solution, in order to reduce the concentration of external solutes and the GV concentration. The dilution step has a double function of decreasing the non-entrapped solute concentration by a factor *b* and simultaneously facilitating GV filtration. Microfiltration will concentrate back the GVs by a factor *β*. As shown by the lipid quantification experiments, however (Fig 2B), the amount of recovered GVs will be low (e.g., 30% or less), due to vesicle rupture on the filter. This reduction of the amount of GVs can be acceptable or not, this depends on the goals of the study (see Discussion).

As a test sample, we prepared 2 mM DOPC/DOPA 7/3 mol/mol GVs from natural swelling in 25 mM Tris-HCl, pH 7.5; calcein was included as hydrophilic membrane-impermeable fluorescent marker. GVs were firstly diluted with the O-solution (*b* = 40), then subjected to microfiltration (*β* = 10). In these conditions, the filtration occurred at [lipids] = 50 μM, while applied Δ*P* was 400 mbar.

**Fig 3:**
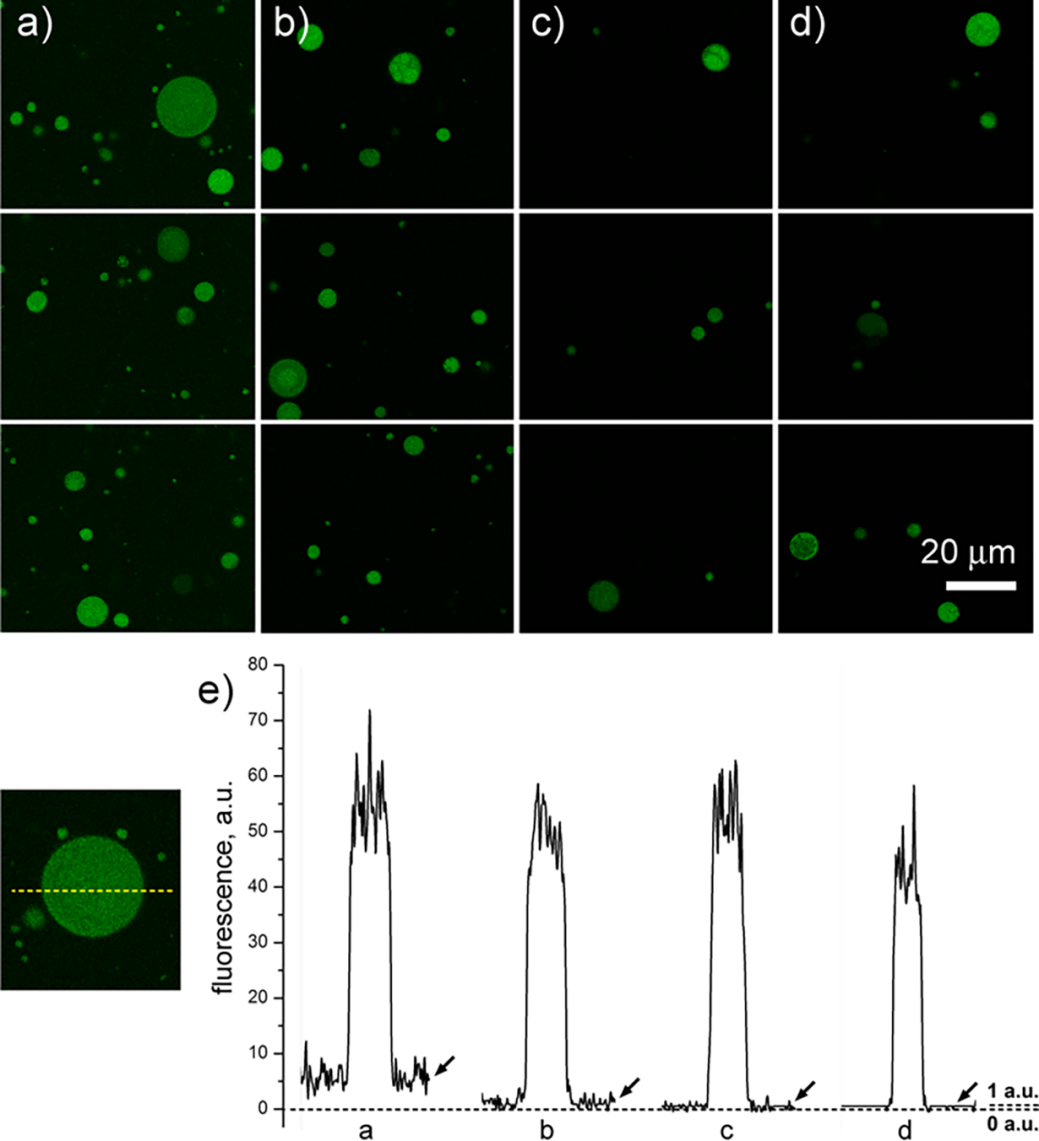
Confocal images and fluorescence profiles of GV samples as obtained by successive microfiltration. (a) Before filtration (after dilution); (b) first retentate; (c) second retentate; (d) third retentate. In (e), typical fluorescence densitometric profiles along GV images are presented, and refer to the (a-d) samples shown on the top. The profiles show the fluorescence intensities along a line drawn over a GV image, see the example on the left. Note that the fluorescence outside vesicles–indicated by arrows–decreases from ca. 6 a.u. (profile ‘a’), to ca. 1 a.u. (profile ‘d’). This latter small value (1 a.u.) cannot be further reduced, as it actually represents the minimal measurable value. Samples consist of calcein-filled DOPC/DOPA 7:3 mol/mol GV in 25 mM Tris-HCl, pH 7.5; see details in Table 1.

The retentate was analysed by means of a confocal microscope, in order to determine the ratio between inner and outer calcein (**1**) concentrations (SNR). As shown in Table 1, entry 1, the ratio was 39:1 in fair agreement with the expectations (40:1). Confocal images of GVs before and after filtration are shown in Fig 3A and 3B, respectively.

**Table 1:** Characterization of DOPC/DOPA 7:3 GV retentates obtained by successive dilution-and-microfiltration steps.

Entry	Average flow rate (μL/s)^*c*^	Lipid concentration (μM)		Recovery rate (%)^*d*^	Signal-to-noise ratio (SNR)	
		Expected	Found		expected	found^*e*^
1^*a*^	43.0	500	200	40 [40]	40:1	39:1
2^*b*^	11.8	200	130	65 [26]	400:1	89:1^*f*^
3^*b*^	9.6	130	80	62 [16]	4000:1	137:1^*g*^

All data refer to filtration operated at Δ*P* = 400 mbar.

^*a*^ *V* = 0.25 mL, *V*_O_ = 9.75 mL, and *V*_ret_ = 1 mL (*b* = 40, *β* = 10)

^*b*^ *V* = 1 mL, *V*_O_ = 9 mL, and *V*_ret_ = 1 mL (*b* = 10, *β* = 10)

^*c*^ the flow rate is high at the beginning of the filtration, then decreases due to partial filter occlusion

^*d*^ the values in square brackets represent the recovery rates referred to 500 μM

^*e*^ the measured values are obtained by analysing GVs in five fluorescence micrographs (values are intended ± 20%), and are limited by the dynamic range of microscope CCD camera, see also Fig 3E

^*f*^ background counts: 1.4 a.u.

^*g*^ background counts: 1.0 a.u.

The lipid concentration in the retentate was measured by the Stewart assay, resulting as 200 μM (expected: 500 μM). A recovery rate of 40% is obtained in this case.

To improve the overall purification procedure, we reasoned that the dilution/filtration processes can be repeated more times. To check the feasibility of this strategy, the retentate obtained after the first filtration was diluted again with O-solution (*b* = 10), and filtered again (*β* = 10). This operation was reiterated twice (Table 1, entries 2 and 3). Three filtrations were passed trough the same filter. In the second and third filtration the flow rates decreases, as expected, because the same partially clogged filter was used. Interestingly, in the second and third filtrations the lipid recovery rates were higher than those of the first filtration (65% and 62% versus 40%), suggesting that the vesicle debris–which covers the filter after the first filtration–reduced the GV’s rupturing during subsequent filtrations. After three dilution/filtration steps, the recovered sample had a lipid concentration of 80 μM which is convenient for most applications. Confocal images of GVs in the second and third retentates are shown in Fig 3C and 3D, respectively.

Although the concentration of the non-entrapped probe is strongly reduced by our procedure, the measured SNR of the second and third retentates (89:1 and 137:1, respectively) did not reach the theoretical values (400:1 and 4000:1, respectively) because the measured fluorescence outside the vesicles is levelled off by the limited instrumental sensitivity. In fact, fluorescent count rates outside the vesicles in the second and third retentates were at the bottom limit of the microscope sensitivity (1.1–1.5 a.u. out of a 0-to-255 levels scale). This is evident in Fig 3E, where a visual representation of the inner/outer probe concentration is given by the fluorescence densitometric profiles along typical GVs. Note that internal fluorescence in the lumen remains approximately constant, whereas the external fluorescence decreases towards a minimal (levelled-off) value.

In conclusion, multiple filtrations greatly enhance the purification efficiency, at the cost of a higher lipid loss and longer filtration times, but never longer than 15 minutes.

### 2.3. Microfiltration at work

#### A case study: The size distribution of ‘primitive cell’ models

Microfiltration can be applied, for example, to the purification of GV samples intended for studies on morphological changes (GV growth, division, fusion, and so on). To this respect it is interesting to measure the size distribution of a GV population before and after microfiltration, in order to check whether such a treatment introduces a bias when size distributions are compared or used as parameter for other deductions. Note that natural swelling typically produces GV populations with a broad size distribution. Narrowly distributed GVs can be prepared by procedures based on the microfluidic preparation of water-in-oil droplets followed by droplet transfer [21], by the differential centrifugation of GVs [22], or by a combination of large-pore extrusion and dialysis [23].

We took the opportunity of our recent investigation on ‘primitive cell’ models based on GVs composed of fatty acids and their simplest glycerol derivatives. In particular, it has been shown that oleic acid, monooleoyl-glycerol, dioleoyl-glycerol, and DOPA (4:1:2:3) form stable GVs that can be considered as the best realistic model for primitive cells, because these four compounds coexist in allegedly primitive phosphorylating conditions [24].

**Fig 4:**
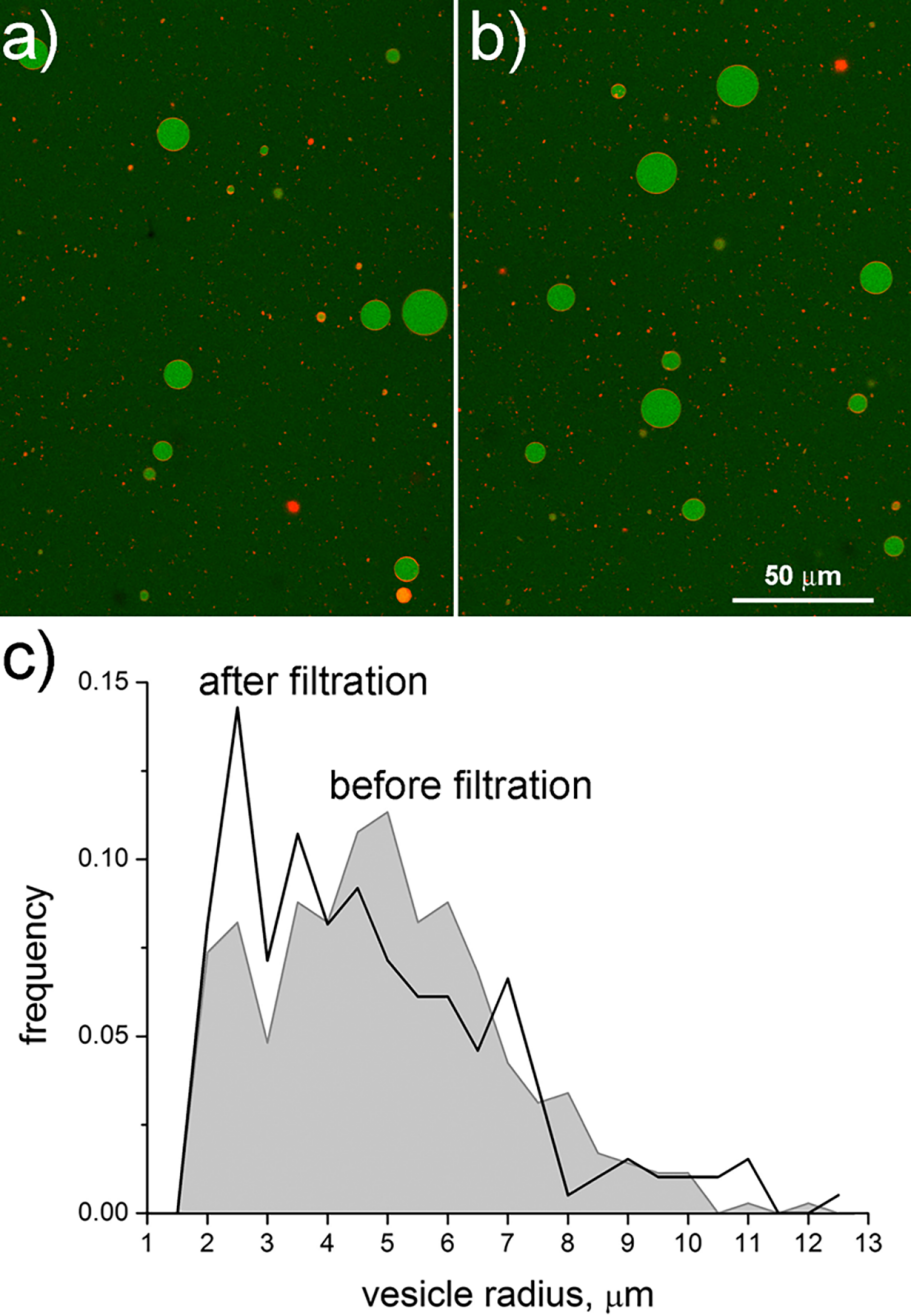
Giant vesicles before and after filtration. GVs prepared by the natural swelling of a mixture of oleic acid, monooleoyl glycerol, dioleoyl glycerol, DOPA (4:1:2:3 molar ratio). Calcein (5 μM) was encapsulated inside the vesicles, while their membrane was stained (by co-hydration) with 0.1 mol% of DOPE-Rh. (a) Raw GVs obtained from film hydration and subsequent dilution with O-solution. (b) Appearance of the retentate after filtration (*b* = 3, Δ*P* = 400, *β* = 10). (c) GV size distribution of the two GV populations (before filtration: grey area; retentate: black line). Statistical analysis detailed in the text.

We have then encapsulated calcein inside these primitive cell models and submitted the resulting population to one filtration step, measuring the vesicle size distribution before and after filtration by confocal imaging and digital image processing. Confocal micrographs (Fig 4A and 4B) show the typical appearance of GVs before and after filtration. The retained sample appears morphologically similar to the original sample. Size distributions are presented in Fig 4C. Their rough comparison suggests that vesicles essentially maintain the same size.

The average GV radius before and after filtration is 5.0 ± 2.0 μm (*n* = 353) and 4.7 ± 2.2 μm (*n* = 196), respectively. The radius difference (– 0.3 μm) is not statistically significant according to an unpaired *t* test with Welch correction, i.e., not assuming equal variances (two-tailed *p* = 0.24). Considering that the size distributions are not Gaussian, non-parametric tests were also carried out. While the Wald-Wolfowitz test gave *p* = 0.28 (i.e., the difference is not significant), the Mann-Whitney U test gave *p* = 0.06, which formally implies a statistically not significant difference at the 95% confidence level, but clearly it suggests caution. On the other hand, the difference was considered statistically significant by the Kolmogorov-Smirnov test (*p* < 0.025).

We conclude that one microfiltration step does not substantially change the GV size distribution. When applied to cases where GV size is a critical parameter, a determination of size distribution may however be necessary, depending also on the specific experimental conditions (one or more microfiltration steps). In particular, the available results indicate a small reduction of size, possibly because of different mechanical stability of small and large vesicles while interacting with the filter. This trend might lead to a reduction of the mean vesicle size after the second or third filtration–to be evaluated case-by-case for accurate conclusions.

A second case study can be found in the S1 File, as supporting information. In particular, the interested readers can find detailed descriptions and comments about the application of three successive microfiltration steps to GVs that have encapsulated a partially hydrophobic solute specifically designed for applications using bioorthogonal strain-promoted click-chemistry (a short mention on the estimation of the partition constant from confocal images is included).

## 3. Discussion

The removal of non-entrapped solutes from vesicle suspensions is an essential step for many applications. To the best of our knowledge, microfiltration was never applied to GV samples, although centrifugal ultrafiltration devices (the type generally used for concentrating proteins) have been used for some operations on conventional submicron vesicles [25], mainly to quantify free solutes or to concentrate vesicles.

Our novel procedure can be advantageously carried out with a very simple filtration apparatus, it does not require any special/expensive device, neither the generation of a density difference between lumen and outside solution, and it is quite fast.

Somewhat expectedly, the filtration rate essentially depends on two major factors, the GV concentration and the pressure difference applied across the filter (Δ*P*). We found that GVs are best filtered at low concentration (e.g., [lipid] = 50–100 μM) and at a moderate Δ*P* (ca. 400–600 mbar). In these conditions, 5 mL of a 50 μM sample are filtered in less than one minute.

We have discovered that filter clogging is the major obstacle of this technique. It reduces both the flow rate through the filter and the number of recovered GVs. On the other hand, we have also observed that filtering GVs over a used (and thus lipid-covered) filter reduces the amount of lipid loss, increasing the recovery rate (Table 1) from ca. 40% to ca. 60%.

Filter clogging never occurs when the applied lipid amount is low (e.g. < 0.5 μmoles), irrespective of the lipid types (DOPC or DOPC/DOPA 7:3) and applied Δ*P* (200–800 mbar). But when one attempts to filter more than 1 μmoles of lipids through a 25 mm diameter filter, clogging is almost certain. This means that a threshold amount of lipid must be overcome to clog the filter. The amount of lipids sticking on the filter correlates with the total amount of lipids applied over the filter (S1 Fig), suggesting that the GVs breakage rate is proportional to the GVs/filter encounter rate and therefore to the volume moved through the filter. For example, data show that up to 0.3 μmoles of lipids can stick to the filter without stopping the filtration. The maximal tolerated amount could be slightly higher, but less than one order of magnitude higher. Considering that the average cross-section of a lipid molecule is around 1 nm^2^, 0.3 μmoles of lipids have a total surface of ca. 1,800 cm^2^, whereas the macroscopic surface of the filter is about 5 cm^2^. The sponge-like microscopic filter structure (see Materials and Methods) explains the fact that such a high amount of lipids can stick to the filter without clogging. In conclusion, microfiltration on nylon filters of the type used in this study safely proceeds until–during the flow–a threshold amount of adsorbed lipid is reached. The threshold value is around 0.1–0.2 μmole/cm^2^.

### 3.1 Possible improvements

From these considerations it is possible to sketch out possible directions for improving the microfiltration methodology. First, using large filters would not change the lipid loss, because this is proportional to the GV/filter encounter rate, but would avoid filter clogging, allowing the filtration of more concentrated GV suspensions. However, operating with larger filter surfaces makes it more difficult to control the volume reduction, and the risk of filtering to dryness increases. Second, a possible way to reduce lipid loss would require a method for filtering the external solution with minimal GV/filter interaction. In principle, this could be achieved if a denser O-solution is employed, so that GVs would mainly stay on the top of the solution due to buoyancy, e.g. glucose in the I-solution, sucrose in the O-solution. Third, one could try to change the filter material in order to reduce its interaction with lipids. A simple possibility is to “prime” the filter with sacrificial small empty vesicles made of same lipids as the GVs. Chemical modifications can also be conceived. The usage of alternative materials is an open issue for future explorations (commercial filters are available in a variety of organic materials like cellulose acetate, cellulose nitrate, PTFE, etc. or inorganic Anopore^®^ filters made of aluminium oxide of highly uniform pore size). We carried out preliminary experiments by using track-etched polycarbonate membranes–those generally employed for vesicle extrusion–which perform similarly to nylon membranes (but lipid loss was not measured). Finally, following the practice of protein filtration, ultrafiltration devices with vertical filters should be tested in order to verify their usability for GVs. Note however that such devices require normally prolonged centrifugation times and high centrifugation speeds.

### 3.2 Comparison with other methods

For GVs two commonly applied methods are dialysis and differential centrifugation. Here we report a rapid purification based on microfiltration. The main features of the two classical methods are commented in Table 2, and compared with those obtained by this study.

**Table 2:** Comparison of GV purification methods.

	Dialysis	Centrifugation	Microfiltration
**Equipment**	Dialysis bag or cassette	Centrifuge	Filter, vacuum pump, pressure regulator
**Duration**	Long (hours)	Short (10–30 min)	Very short (< 10 min)
**Recovery rate**	Medium-high	Medium (> 50%)	Low (< 50%)
**Essential requirement(s)**	None	Density difference (use of sugars)	Low lipid concentration
**Main advantage**	Mild conditions	Easy	Easy and fast
**Main drawback**	Slow	Need of a density difference	Low recovery rate

In the dialysis method, crude GVs are enclosed in a dialysis bag suspended in a large volume of O-solution. The chemical components of the I- and O-solutions that freely diffuse through the dialysis membrane slowly reach their equilibrium concentration. Purified GVs are recovered from the dialysis bag. A large volume of O-solution is required, and the required long time (hours) restricts this purification method to those solutes that are not massively released from GVs in the allotted time, otherwise the intravesicle solute concentration would also decrease. Recently, the use of home-modified small-volume commercial dialysis cassettes for exchanging the solution outside vesicles has been reported [26]. This method is particularly useful when high-cost solutions must be exchanged with vesicle suspensions.

In the differential centrifugation method, the establishment of a density difference is required. This is typically done by including sucrose in the I-solution and glucose in the O-solution. GVs are thus diluted with O-solution and centrifuged. Vesicles are recovered from the bottom of the tube. However, recovering the GVs pellet means–in practical terms–collecting a not-so-small supernatant volume, which contains the non-entrapped solute at the same high concentration as the initial solution. This implies that several centrifugations are often needed to purify GVs at a certain requested extent, with consequent lipid loss and spent time.

It should be mentioned that the dialysis and centrifugation, together with microfiltration, give free-floating GVs. If this is not a requirement, another easy procedure to “purify” GVs from the non-entrapped material is the GVs anchoring to a solid support (*e*.*g*., the microscope glass slide) followed by a simple exchange of the external solution. A convenient method is based on biotinylated GVs obtained by including a small amount of biotinylated lipids during their preparation. The resulting GVs can be anchored to a biotinylated surface (for example obtained by adsorption of biotinylated albumin to the support) by means of streptavidin linking [27,28].

### 3.3 Possible applications of the GV microfiltration technique and device

Our interest in GV microfiltration was initially triggered by looking at some modern techniques for a cell culture assay, such as the two-chamber devices for cell transmigration assay, co-culture, or similar applications (S2 Fig). The ‘floor’ of top chamber is a filter that allows the passage of small molecules (not whole cells) from the top to the bottom chamber, and vice versa. We reasoned that a similar architecture could be used for operating on a GV population. In particular, GVs placed (or ‘grown’) on the filter could be treated with various substances, in order to induce morphological transformation, chemical reactions, or any other behaviour of interest. The semi-permeable filter would facilitate, therefore, the removal of unreacted substances, GV fragments, or any other by-product. In other words, GVs–which are intended in most studies as cell models–could be manipulated as biological cells with the help of similar devices. This scenario could be implemented either for individual GVs, considering a differential stability of diverse types of GVs within a population, or for GV communities (‘colonies’ [29,30]).

While these intriguing approaches have not been developed yet, here we have clarified some aspects of GV microfiltration with the explicit intention of presenting a novel and general purification method, which should be of broader interest. Last but for us by no means least, the loss of GVs through microfiltration simulates a natural selection process that could be used to select ‘fitter’ GVs grown from complex mixtures [31].

## 4. Concluding remarks

GVs are emerging cellular models employed in biophysics, biochemistry, origin of life studies, systems chemistry, synthetic biology. Their preparation methods have been recently expanded, thanks to the introduction of the droplet transfer method and microfluidic-based devices. However, methods for their post-formation processing, like the elimination of free non-encapsulated solutes, have not similarly advanced. Here we report a novel, yet simple and cheap, methodology based on microfiltration. Such a method enriches the experimental toolbox for GV manipulations, its rapidity being the principal advantage. We have applied GV microfiltration to two case studies, in order to show opportunities and limitations. For example, we have introduced a molecular device (a clickable fluorescent probe) intended for the use of GVs to screen libraries of compounds. Moreover, the study paves the way to future approaches where GVs are submitted to *in situ* treatments and survival (selective retention) of the fitter.

## 5. Experimental section

### 5.1 Abbreviations

DOPA: 1,2-dioleoyl-*sn*-glycero-3-phosphatidic acid;

DOPC: 1,2-dioleoyl-*sn*-glycero-3-phosphatidylcholine;

DOPE-Rh: 1,2-dioleoyl-*sn*-glycero-3-phosphatidyletanolamine-*N*-(lissamine rhodamine B sulfonyl) ammonium salt.

### 5.2 Materials

DOPA, DOPC, DOPE-Rh were purchased from Avanti Polar Lipids Inc. (Alabaster, AL); all other chemicals and solvents were from Sigma-Aldrich and were used without purification. Nylon filters were from GE Healthcare Life Sciences (Whatman nylon membrane filters, 0.2 μm pores, 25 mm diameter, filter thickness 60–120 μm; Cat. No. 7402–002, Lot. No. G9967297). Typical electron micrographs of nylon filters can be found at the URL

(a) https://www.membrane-solutions.com/nylon_disc_membrane.htm

(b) http://www.advantecmfs.com/filtration/membranes/mb_nylon.php

I-solutions were composed of (a) 5 μM calcein, 5 mM Na-bicine (pH 8.5); or (b) 5 μM calcein, 25 mM Tris-HCl, pH 7.5. O-solutions were composed of (a) 5 mM Na-bicine (pH 8.5); or (b) 25 mM Tris-HCl pH 7.5. Note that, although not strictly necessary, sometimes the I-solution and O-solution included also 200 mM of sucrose and glucose, respectively.

### 5.3 Methods

#### 5.3.1 GV preparation

GVs were prepared by the natural swelling method. A known amount of lipids, dissolved in chloroform, was placed in a round-bottom thick-wall 10 mL glass tube (diameter ca. 1.5 cm), and the solvent was evaporated under reduced pressure by a Büchi Rotavapor, and further dried in high vacuum (1–10 mbar) for 15 minutes. When needed, 0.1 mol% DOPE-Rh was included in order to stain the vesicle membrane with red fluorescence. The dried film was hydrated without shaking for 15 hours at 25°C with I-solution that included 5 μM calcein as water-soluble membrane impermeable green fluorescent fluorophore. When needed, the resulting GVs were diluted with the corresponding isotonic O-solution.

Primitive cell-like compartments made of oleic acid, monooleoyl glycerol, dioleoyl glycerol, and DOPA (molar ratios 4: 1: 2: 3) were prepared in 25 mM Tris-HCl (pH 7.5) by the natural swelling method. More details on this vesicle system have been reported elsewhere [24].

#### 5.3.2 GV microfiltration

The filtration apparatus is shown in Fig 1B. It consists of a thick-walled vacuum Erlenmeyer flask connected to a vacuum pump (BrandTech Vacuubrand). Note that, because the required vacuum is moderate, a Venturi vacuum water pump can be also used. A funnel-shaped Teflon filter holder is placed above the flask (GE Healthcare). A pressure regulator (Vakuum-Regler, Glas Keller, Basel, CH) is inserted between the flask and the pump allowing control the pressure exerted by the pump (*P*_pump_) below the filter. The applied pressure difference Δ*P* (mbar) across the filter is equal to *P*_atm_−*P*_pump_ ≈ 1000 mbar–*P*_pump_. A determined volume of GVs was applied over the filter. Filtration was started by operating on pump, and continued until desired. Care should be taken to avoid that the whole applied volume is sucked down in the flask. If needed, a Falcon™ tube can be placed under the funnel tip to collect the filtrate.

#### 5.3.3 Lipid quantification

When required, the DOPC concentration was determined by the Stewart assay [20], with minor modifications. In our protocol, a DOPC GV suspension (100 μL) was thoroughly mixed with chloroform (600 μL) and the assay solution (0.1 M FeCl_3_ 6H_2_O, 0.4 M NH_4_SCN, 0.4 M KCl; 150 μL). After phase separation, the organic phase was placed in a 1 cm-path length spectrophotometric cuvette to measure the absorbance at 470 nm. The instrument was blanked by a negative control sample; DOPC vesicles of known concentration have been used for calibration. Considering a noise of about 0.003 absorbance units (a.u.), and a response of about 1.4 a.u. cm^-1^ mM^-1^, the detection limit can be estimated as ca. 6 μM (concentration refers to the 100 μL sample).

#### 5.3.4 GV observation by confocal microscopy and image analysis

Crude GVs (or diluted with O-solution when needed) were placed in a micro-well plate (code No. 81821, ibidi GmbH, Martinsried, Germany) and observed after ca. 10–15 minutes. Each circular micro-well has a diameter of 5 mm and can hold a maximum of about 35 μL. Samples have been observed by means of a Leica TCS SP5 confocal microscope (Leica Microsystems, Wetzlar, Germany), using a 63× oil immersion lens. Calcein and DOPE-Rh have been detected by employing standard settings of the Leica software, but employing the sequential acquisition mode.

Digital image processing and analysis was carried out using ImageJ [32]. To measure internal or external fluorescence, a ROI was selected inside or outside the GVs projection in the digital image, and the average pixel luminosity was measured. Similarly, the software provides the pixel luminosity along a line drawn on the image, which is useful for illustrative purposes. Note that images have been recorded at 8 bits colour depth (256 levels, from 0 to 255), and that the minimal luminosity value is around 1, even for non-fluorescent samples. To determine the size distribution, a threshold was applied to the 8-bit images in order to convert them into 1-bit images. The latter were processed by the built-in object recognition algorithm allowing the identification of brighter circular objects over a darker background. The ROI map was then applied to the original 8-bit images and vesicle parameters were measured (projected area and fluorescence). Shape descriptors (circularity > 0.6, and absence of holes) were used to discard bad-quality vesicles. From the measured circular projected area, under the hypothesis that it corresponds to the great circle of the spherical vesicle, the vesicle radius was calculated.

## Supporting information

S1 File Case study: Synthesis and encapsulation of BCN-Fluo inside GVs. The file includes the synthesis and the chemical characterization of BCN-Fluo, a non-commercial partially hydrophobic solute for click-chemistry, its incorporation inside GVs, description of the microfiltration results (three filtration steps), discussion of the results, and the estimation of the BCN-Fluo membrane partition constant from fluorescent confocal images.
(PDF)

S1 Fig Estimating the amount of lost lipid. Variation of the amount of lost lipid (stacked on the filter) with the amount of applied lipids (data refer to filtrations carried out at Δ*P* = 400 mbar).
(TIF)

S2 Fig A two-chamber device. Schematic representation of a two-chamber device (sometimes referred to by the tradename Transwells®, Corning) and its hypothetical use for experiments involving GVs (intravesicle reaction, GV transformation, selection experiments, GV growth, etc.).
(TIF)
