# Supplementary material for: Rapid purification of giant lipid vesicles by microfiltration
Source: PLoS One. 2018 Feb 16;13(2):e0192975. doi: 10.1371/journal.pone.0192975 (PMC5815610; doi:10.1371/journal.pone.0192975)
Supplement: S1 File — The file includes the synthesis and the chemical characterization of BCN-Fluo, a non-commercial partially hydrophobic solute for click-chemistry, its incorporation inside GVs, description of the microfiltration results (three filtration steps), discussion of the results, and the estimation of the BCN-Fluo membrane partition constant from fluorescent confocal images. (PDF) [file pone.0192975.s001.pdf]

S1 File

Case study: Synthesis and encapsulation of BCN-Fluo inside GVs

Rapid purification of giant lipid vesicles by microfiltration

Dimitri Fayolle,[1] Michele Fiore,[1] Pasquale Stano,[2†*] and Peter Strazewski[1*]

[1] Institut de Chimie et Biochimie Moléculaires et Supramoléculaires, Université de Lyon, Claude Bernard Lyon 1, 43 bvd du 11 Novembre 1918, F–69622 Villeurbanne Cedex, France

[2] Department of Sciences, Roma Tre University, Viale. G. Marconi 446, I-00146 Rome, Italy

[†] Current address: Department of Biological and Environmental Sciences and Technologies (DiSTeBA), University of Salento, Ecotekne, I-73100 Lecce, Italy

Corresponding Authors: pasquale.stano@unisalento.it; strazewski@univ-lyon1.fr

Table of Content

**1**	**Encapsulation of BCN-Fluo inside GVs and application of the microfiltration technology**	**Page 02**
**2**	**Methods**	**Page 06**
	**2.1 Materials and instrumentation**	**Page 06**
	**2.2 Discussion on the preparation of the BCN-Fluo (2)**	**Page 06**
	**2.3 Encapsulation of BCN-Fluo inside GVs**	**Page 16**
	**2.4 Estimation of BCN-Fluo partition constant**	**Page 16**
**3**	**References**	**Page 19**

Abbreviations

BCN: bicyclo[6.1.0]non-4-ynyl

1. Encapsulation of BCN-Fluo inside GVs and application of the microfiltration technology

In order to test the effectiveness of GV purification in real cases, we have applied it to a case study of our interest, that is, the encapsulation of a fluorescent 'clickable' probe inside GVs.

Click chemistry encompasses one-pot, water-compatible, high-yield reactions that serve to bind two specific reaction partners under mild and extremely chemoselective conditions. Among the various clickable reactants, we focused on copper-free 'strain-promoted azide-alkyne cycloaddition' (SPAAC), which is a reaction based on cycloalkyne derivatives and azide moieties (Fig S1.1a). SPAAC reactions are, in addition to being fast and chemoselective, also 'bio-orthogonal' meaning that, in conjunction with hydrosoluble organic azides, they can be applied in aqueous solution, in GVs, in cells, partly even *in vivo*.[1] The fluorescent probe BCN-Fluo (**2**, Fig S1.1b) was obtained from commercial bicyclo[6.1.0]non-4-yn-9-ylmethanol (BCN-OH).[2] We linked BCN-OH to fluoresceinamine through a carbamate bond to form a BCN-fluorescein derivative (BCN-Fluo). The synthesis of **2** and structure of the synthetical intermediates are shown in Fig S1.1.

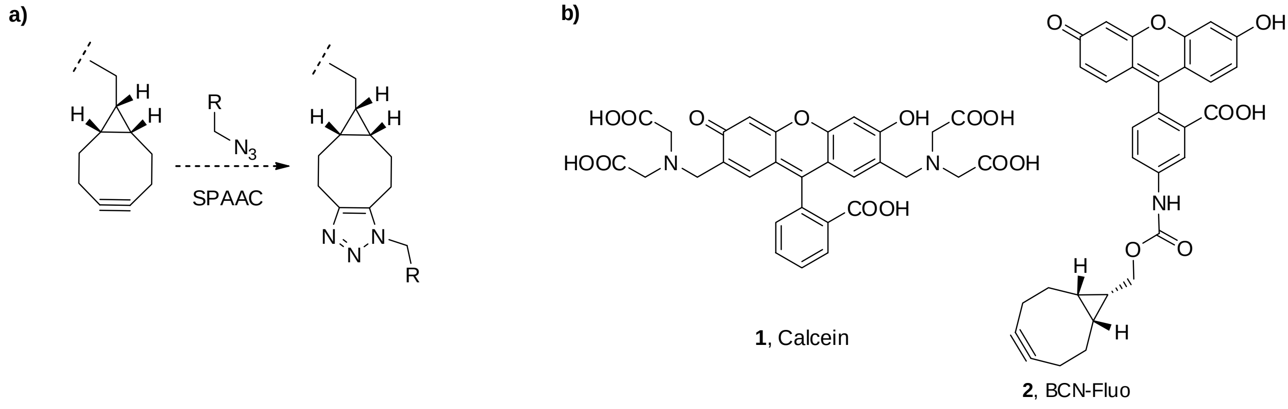

Fig S1.1. Fluorophores used in this study. (a) Scheme principle of a click reaction between a strained cycloalkyne (BCN) and an organo-azide (RCH_2N_3). (b) The structure of calcein (**1**) and of the clickable probe BCN-Fluo (**2**).

The encapsulation of BCN-Fluo (**2**) inside GVs allows for the construction of a cell-like microreactor for screening libraries of azide-derivatized molecules with respect to membrane permeability. After incubation of GVs containing BCN-Fluo with the library of interest, only those molecules capable of permeating the vesicle membrane will be found covalently linked to the BCN-Fluo, and next identified. The fluorescein moiety is a useful tag for monitoring the intra-vesicle location of BCN adducts, as well as for applying fluorescence-based high-throughput analytical or preparative methods (flow cytometry, FACS) in other experimental steps.

DOPC GVs (0.5 mM) were prepared by the natural swelling method employing, as I-solution, 40 μM BCN-Fluo and 5 mM Na-bicine (pH 8.5). The presence of BCN-Fluo in the I-solution did not significantly affect the outcome of lipid hydration, as qualitatively estimated by direct observation of the resulting GVs by confocal microscopy. However, we noted that the partially lipophilic character of BCN-Fluo results in a partitioning of the probe both in the GVs aqueous lumen and in the lipid membrane (Fig S1.2).

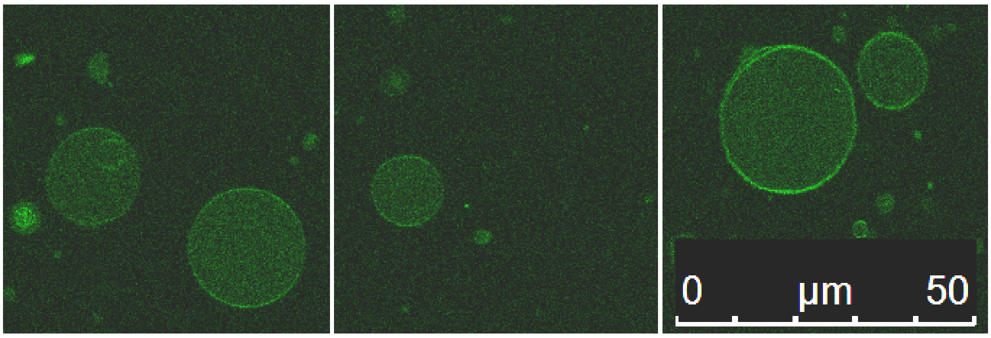

Fig S1.2. Confocal microscopy images of BCN-Fluo-containing GVs

The lipid/aqueous phase BCN-Fluo partition coefficient has been estimated by quantitative analysis of confocal fluorescence images (details in the experimental part below), following a procedure, introduced by us elsewhere,[3] resulting in a partitioning constant $K_x \approx 1.5 \times 10^5$ in favour of the membrane (log$P$ ≈ 5.2), *i.e.*, $\Delta G°_{partition} = -7.1$ kcal/mol at 298 K. This value fits well with the hypothesis that BCN-Fluo interacts with the phospholipid membrane by cyclooctyne moiety insertion (see electronic supplementary information for a discussion).

After preparation, GVs were first diluted (b = 40; [DOPC] = 125 μM) and then purified from the non-entrapped BCN-Fluo by applying three successive filtrations (as described in the main text). The experimentally measured SNR of the GVs after three filtrations is 65:1 (theoretically 4000:1), but – as already mentioned – the limited dynamic sensitivity range of the confocal digital camera levels off the fluorescence value of the dark background. Typical confocal images of BCN-Fluo-containing GVs, before and after one, two, and three filtrations, are shown in Fig S1.3. An illustration of how successive filtrations improve the SNR is also reported, evidencing that the effectiveness of the whole process is based on multiple dilution-filtration-concentration passages. The whole procedure took about 10 minutes to be completed, and did not require any advanced skills. These experiments show how facile and rapid the removal of non-entrapped solutes by micro-filtration of GVs is, provided that the initial lipid concentration is not too high.

A caveat at this point: after the GV purification, the residual extra-vesicle BCN-Fluo concentration in the O-solution is expected to be 10 nM. This value might be satisfying or not, depending on the intended GV's usage. It should be considered that, indeed, even if the ratio between the internal/external BCN-Fluo concentrations is high (4000:1), the ratio between the *number* of BCN-Fluo molecules inside and outside vesicles is not so high. This is because the ratio between the GVs volume and the external volume is generally very low. Using a volume ratio estimate of 1:100 (sum of GV volumes / external volume) the ratio between the overall number of probe molecules (inside/outside GVs) is around 40:1, meaning that 2.5% of the probe is still outside the vesicles. Therefore, depending on the experimental scopes, it becomes important to evaluate whether the further purification steps are required or not.

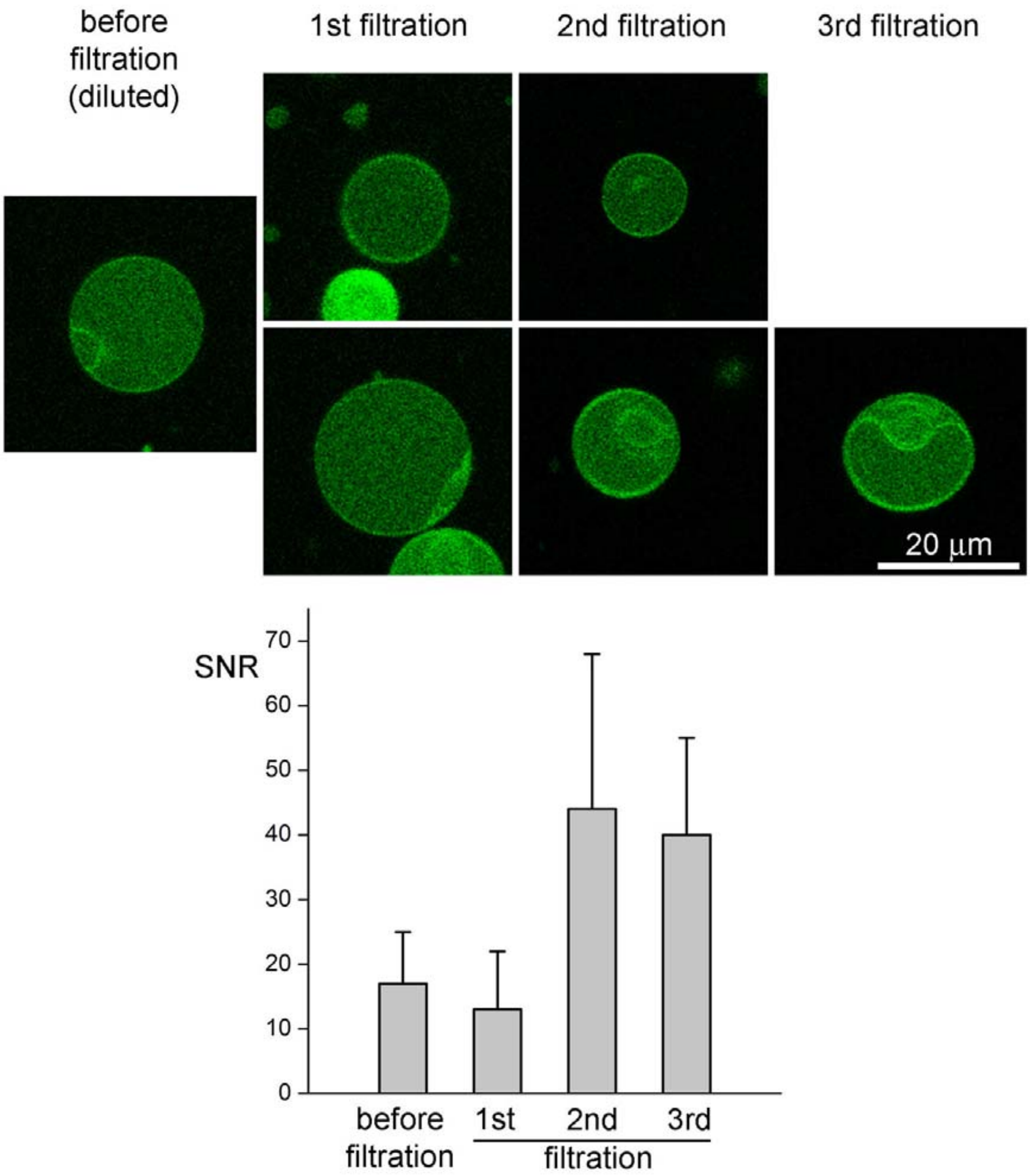

Fig S1.3. Characterization of BCN-Fluo-filled DOPC GV retentates as obtained after successive filtration steps. Top: confocal images of representative GVs before and after three filtrations, the background fluorescence decreases after each step; note that BCN-Fluo accumulates at the membrane. Bottom: SNR of the GVs as obtained by quantitative image analysis. Bars refer to average ± standard deviation of *n* GVs (before filtration: *n* = 9; after first, second, and third filtration: *n* = 41, 32, and 15, respectively).

2. Methods

2.1 Materials and instrumentation

Oleic acid was purchased from Sigma-Aldrich and TCI-Europe and used without further purification. DOPA, DOPE, DOPC and DOPE-Rh were purchased from Avanti Polar Lipids, Alabaster AL (USA). All the other reagents and solvents were purchased from Sigma-Aldrich, Fischer scientific and TCI Europe and were used without further purification. HPLC solvents were purchased from Thermo-Fisher Scientific (mass spectrometry grade).

Thin-layer chromatography (TLC) was carried out on aluminium sheets coated with silica gel 60 F254 (Merck). TLC plates were inspected by UV light (λ = 254 nm) and developed by treatment with a mixture of 10% H_2SO_4 in EtOH/H_2O (1:1 *v/v*), $KMnO_4$ 10% solution or the *Pancaldi* reagent ($(NH_4)_6MoO_4$, $Ce(SO_4)_2$, H_2SO_4, H_2O) followed by heating.

ESI–MS and UPLC–HRMS analyses were performed on a Bruker Impact II equipped with a hybrid quadrupole type mass spectrometer.

NMR spectra were recorded in $CDCl_3$ and CD_3CN on a Bruker Avance 300MHz spectrometer at 300 for ^{1}H nuclei and 75 for ^{13}C nuclei and on a Bruker Avance 400 MHz for ^{1}H nuclei and 100 for ^{13}C nuclei. Chemical shifts of solvents ($CDCl_3$: δ_H = 7.26 and δ_C = 77.23; CD_3CN: δ_H = 1.94 and δ_C = 118.89) served as internal references. Signal shapes and multiplicities are abbreviated as br (broad), s (singlet), d (doublet), t (triplet), q (quartet), quint (quintet) and m (multiplet). Where possible, a scalar coupling constant *J* is given in Hertz (Hz).

2.2 Discussion on the preparation of the BCN-Fluo (2)

The analysis of compartmented systems is a challenging issue. Biomolecules are not visible under natural light because of their size, whereas GVs are micron-scale objects. Using microscopy to monitor the behavior of biomolecules in those cell-like micron-scale objects is hence adapted. In particular, in the perspective of studying the ability of prebiotic cell-like compartments to encapsulate primitive forms of bioactive nucleic acids or proteins (i.e. possessing catalytic properties), one convenient way is to use fluorescent tags. This strategy has been studied for a long time in many fields and lead to the development of various fluorescent probes that can be attached to biomolecules using clickable biorthogonal fragments.[1] However, to develop reactions that are suitable for in-vivo use is highly challenging. The presence of endogenic nucleophiles, such as proteins, carbohydrates and other biomolecules, represent one major limitation. Water-compatibility, the impossibility to use catalysts and metals, temperature limitation and toxicity concerns brings the challenge one step higher. The Staudinger ligation between a phosphine and an azide group group, for example, requires an intramolecular electrophilic trap and thus an additional

sterically hindering moiety.[4] Furthermore, azide groups that have been tagged around cell membranes were partially reduced to amines through the classical Staudinger reaction, instead of undergoing chemical ligation.[5]

Reagents undergoing a rapid cycloaddition reaction are better candidates to satisfy the above-mentioned requirements. Their particular reaction mechanism, the possibility to fine-tune their reactivity through the usage of slightly different derivatives and the use of fragments such as azido groups or small unsaturated cycles (cyclopropenes) that do not occur in living organisms, make them ideal for biorthogonal ligation inside cell-like compartments such as GVs. Remarkably, the team of Bertozzi has developed a range of strained alkynes such as cyclooctynes,[6] biarylazacyclooctynones,[7] and thiacycloalkynes[8] that can efficiently be clicked to azides in water and without the need of copper catalysts, thus adapting the widely used Huisgen reaction to the requirements of living systems. The success of those reagents and their decently easy access lead to the commercialization of certain moieties (the (1*R*,8*S*,9*s*)-bicyclo[6.1.0]non-4-yn-9-ylmethanol, often referred to as BCN, Scheme S1.1).
The coupling of commercial clickable fragments such as commercial BCN-OH (**3**) to endogenous nucleophiles is precedented, with *N,N'*-disuccinimidyl carbonate (Su_2O, **4**) as a source of electrophilic carbonyl.[9] The hydroxy group was successfully activated with **4** to give the stable intermediate **5** (BCN-Fluo, Figs S1.4-S1.5) in very good yields. Compound **5** was further coupled with amino-fluorescein (**6**) in the presence of diisopropylethylamine (DIPEA) to give **2** in reasonable amounts (Scheme S1.1).

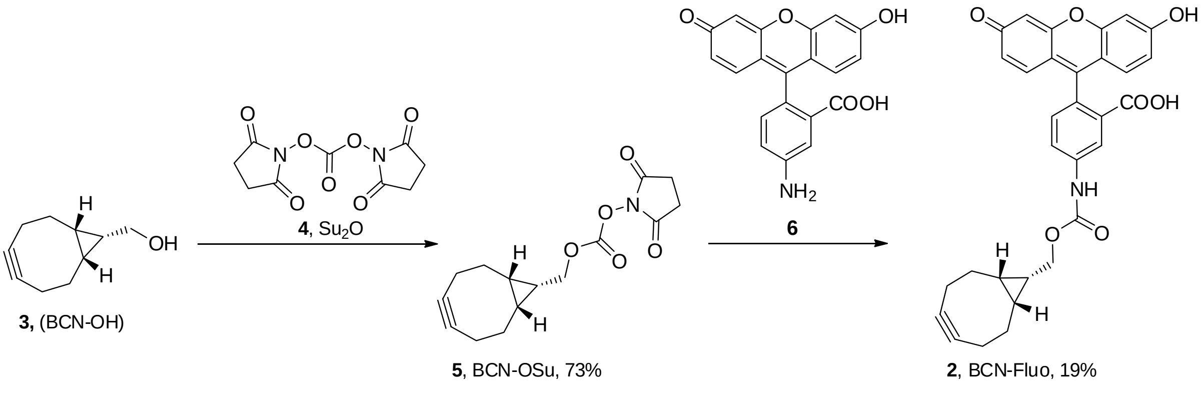

Scheme S1.1. The total synthesis of the fluorescent tag BCN-Fluo (**2**) from commercial BCN-OH (**3**).

Synthesis of (1*R*,8*S*,9*s*)-bicyclo[6.1.0]non-4-yn-9-ylmethyl (2,5-dioxopyrrolidin-1-yl) carbonate (5)

BCN-OH (**3,** 72.6 mg, 0.48 mmol) and *N,N'*-succinimidyl carbonate (**4,** 256 mg, 1.00 mmol) were dissolved in anhydrous acetonitrile (2.5 mL) and placed under argon. Dry triethylamine (200 µL, 1.46 mmol) was added and the mixture was stirred overnight at room temperature. The crude mixture was extracted with AcOEt/Et_2O (80 mL 1:1, *v/v*), washed with water (6 x 15 mL), brine (2 x 5 mL), dried over $MgSO_4$ and evaporated under reduced pressure. The residue was purified by flash column chromatography, eluting with AcOEt/hexanes (1:2, *v/v*) to give the title compound **5** (102 mg, 73%) as a white powder. NMR data are in accordance with previous report elsewhere.[2] ^{1}H NMR (300 MHz, $CDCl_3$): δ = 3.66 (d, *J* = 7.9 Hz, 2H, C(10)H_2), 2.77 (s, 4H, C(13)H_2 and C(14)H_2), 2.20 (m, 6H, 3 x CH_2, C2, C3 and C6), 1.57 (m, 2H, CH_2, C7), 1.27 (m, 1H, C(9)H), 0.89 (m, 2H, C(1)H and C(8)H) ppm; ^{13}C NMR (75 MHz, $CDCl_3$) δ = 168.8 (C12 and C15, 2 x C=O), 151.6 (C11, O_2C=O), 98.7 (C4 and C5, C≡C), 70.3 (C10), 28.9 (C3 and C6), 25.5 (C13 and C14), 21.3 (C2 and C7), 20.7 (C1 and C8), 17.1 (C9) ppm.

Synthesis of 5-((((1*R*,8*S*)-bicyclo[6.1.0]non-4-yn-9-ylmethoxy)carbonyl)amino)-2-(6-hydroxy-3-oxo-9,9a-dihydro-3H-xanthen-9-yl)benzoic acid (2)

BCN-OSu (**5**, 30 mg, 0.10 mmol) and commercial fluoresceinamine (**6**, 70 mg, 0.20 mmol) were dissolved in dry DMF (1 mL). DIPEA (35 µL, 0.20 mmol) was added and the mixture was stirred overnight. The crude mixture was then extracted with Et_2O/AcOEt (1:1, 2 x 10 mL), washed with water (6 x 10 mL), brine (2 x 10 mL). The collected organic phases were dried over $MgSO_4$ and concentrated under vacuo. The crude mixtures were purified by flash chromatography with AcOEt/cyclohexane (2:8 to 8:2 *v/v*) to give **2** (9 mg, 19 %) as a yellow wax. ^{1}H NMR (400 MHz, CD_3CN) δ = 7.57 (s, 1H, N<u>*H*</u>), 7.18 (dd, *J*= 1.6, 1.2, 1H, CH_{Ar}), 7.12 (dd, *J*=2.1, 0.4, 1H, CH_{Ar}), 7.04 (dd, *J*=8.3, 2.2, 1H, CH_{Ar}), 6.96 – 6.94 (m, 2H, CH_{Ar} and CH_{Ar}, 6.93 (dd, *J*=8.3, 0.4 1H, CH_{Ar}), 6.77 (d, *J*=8.7, 1H, CH_{Ar}, 6.74 (d, *J*=2.5, 1H, CH_{Ar}), 6.62 (dd, *J*=8.7, 2.5, 1H, CH_{Ar}), 4.72 (s, 2H, 2 x OH), 4.39 (d, *J*=9.3, 2H, C(10)H_2), 2.36 – 2.16 (m, 4H, C(3)H_2 and C(6)H_2 and HDO as br s 2.20 ppm), 1.73 – 1.57 (m, 4H, C(2)H_2 and C(7)H_2), 1.48 (quint, 1H, *J*=8.9, C(9)H), 1.30 (s, 1H, OH), 1.08 – 0.98 (m, 2H, C(1)H and C(8)H); ^{13}C NMR (101 MHz, CD_3CN) δ = 171.6 (C=O), 170.4 (-<u>C</u>OOH), 159.8 (COH), 154.0 (*C*H_{Ar}), 153.2 (*C*$_{Ar}$), 153.0 (O<u>C</u>=ONH), 152.6 (*C*H_{Ar}), 151.0 (*C*$_{Ar}$), 142.2 (*C*$_{Ar}$), 130.3 (*C*H_{Ar}), 130.2(*C*H_{Ar}), 128.6 (2 x *C*$_{Ar}$), 125.2 (*C*H_{Ar}), 123.0 (*C*H_{Ar}), 119.0 (*C*$_{Ar}$), 118.1 (*C*H_{Ar}), 113.5 (*C*H_{Ar}), 110.6 (*C*H_{Ar}), 108.0 (*C*H_{Ar}), 103.3 (*C*$_{Ar}$), 99.6 (C4 and C5, <u>C</u>≡<u>C</u>), 68.4 (C10, <u>C</u>H_2), 29.6 (C3 and C6, 2 x <u>C</u>H_2), 21.7 (C2 and C7, 2 x <u>C</u>H_2), 21.1 (C1 and C8, 2 x <u>C</u>H), 18.0

(C9, C̲H); HRMS $[M+H]^+$ calcd for $C_{31}H_{26}NO_7$ 524.1704, found 524.1686 (3.5 ppm); Residual solvent (25%): AcOEt: 4.09 (q, 2H, *J*=7.1), 2.00 (s, 3H), 1.23 (t, 2H, *J*=7.1).

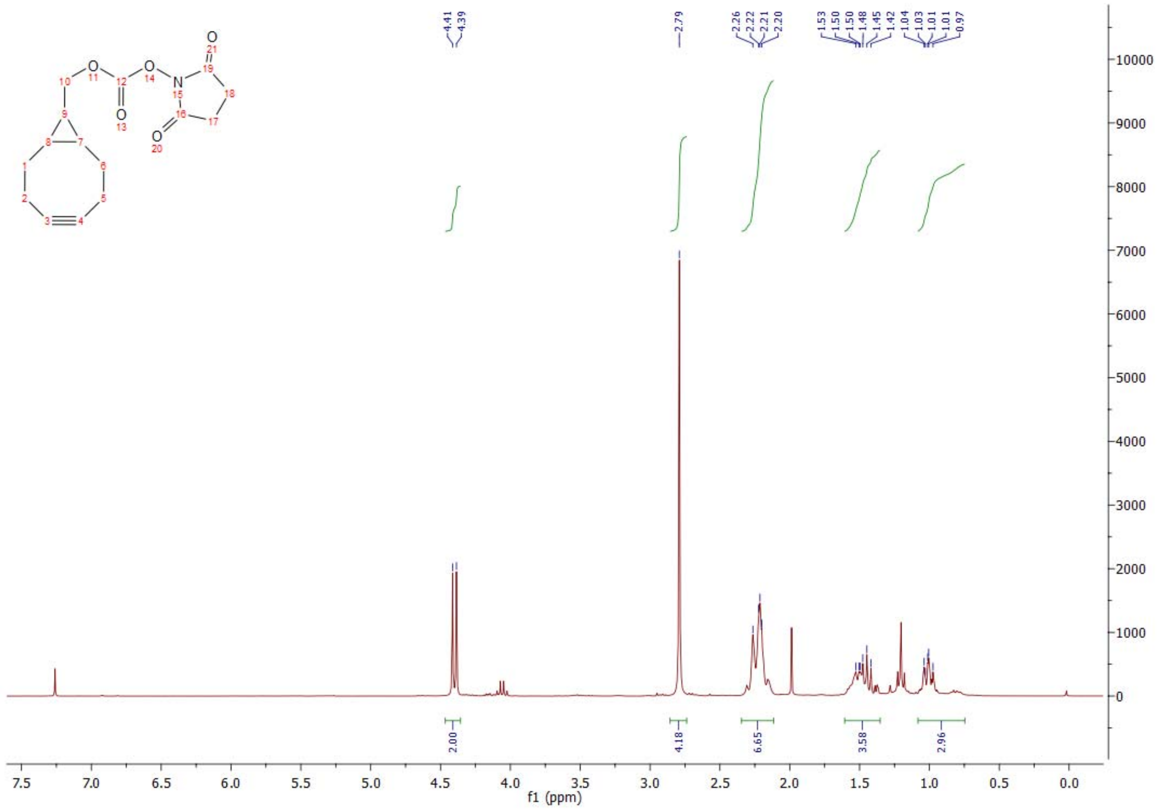

Fig S1.4. ^{1}H NMR (300 MHz, $CDCl_3$) of compound **5**.

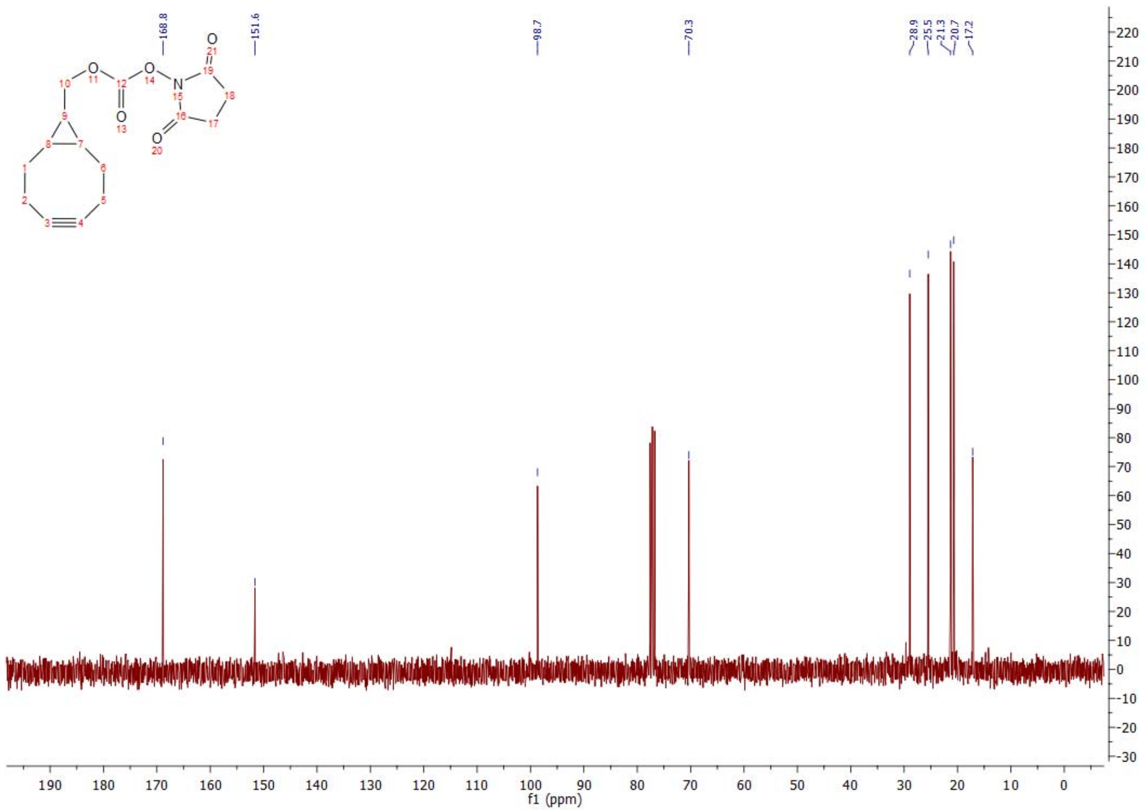

Fig S1.5. ^{13}C NMR (75 MHz, $CDCl_3$) of compound **5**.

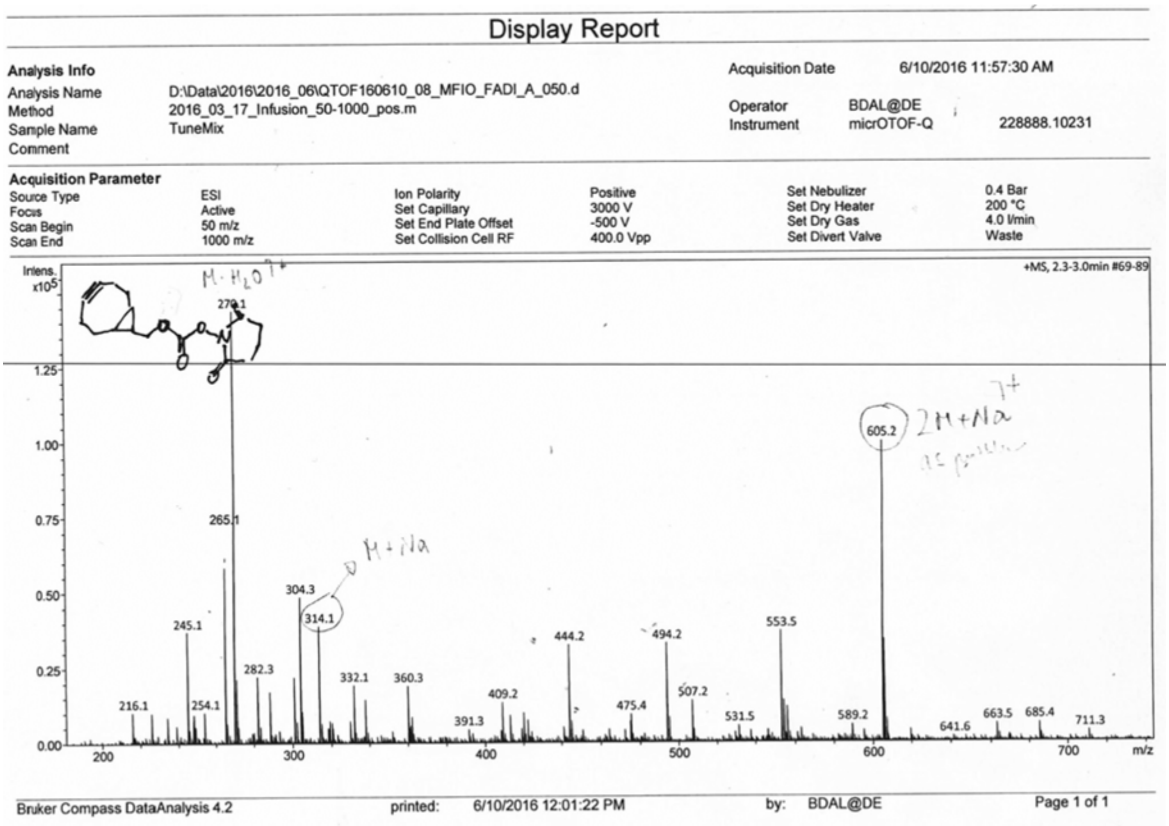

Fig S1.6. ESI-MS (positive ion mode) of compound **5**.

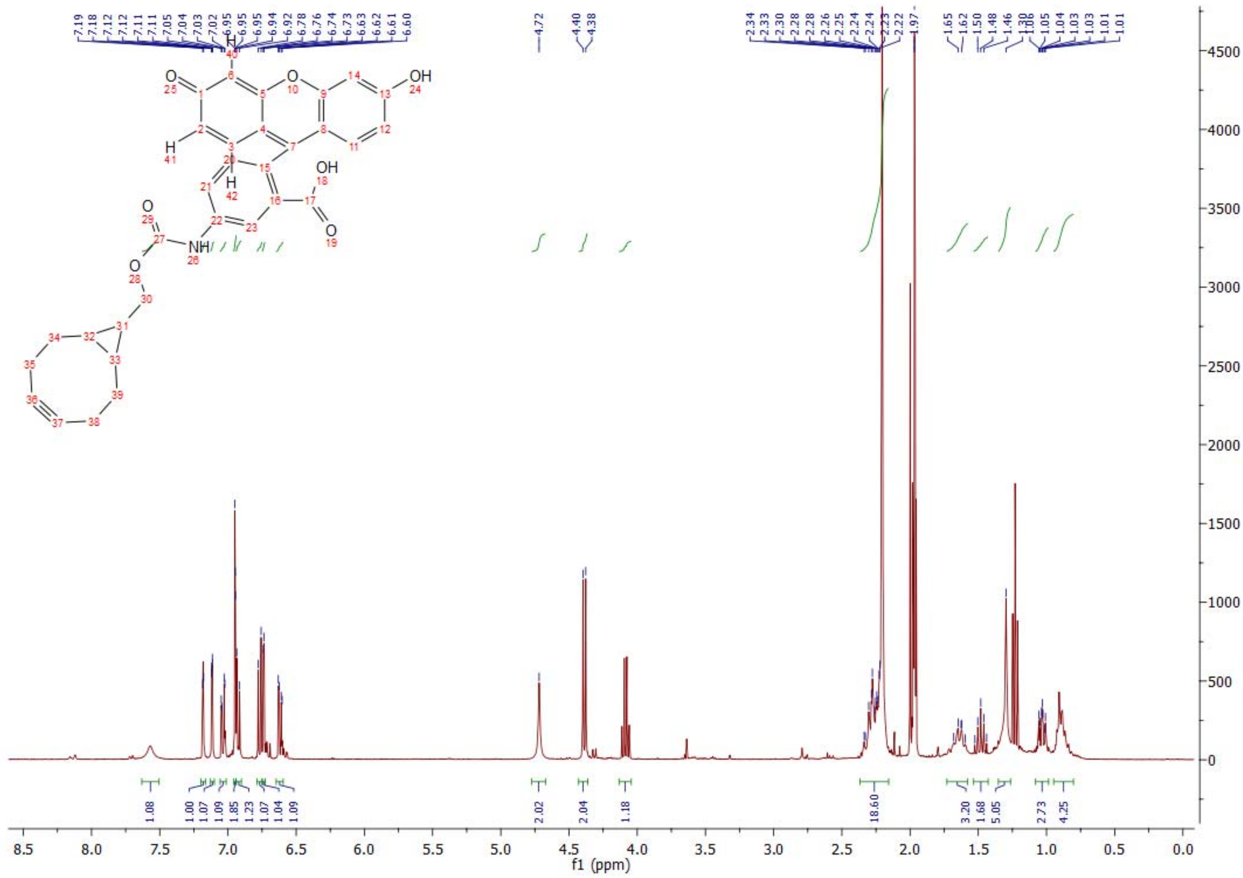

Fig S1.7. ^{1}H NMR (400MHz, CD_3CN) of compound **2**.

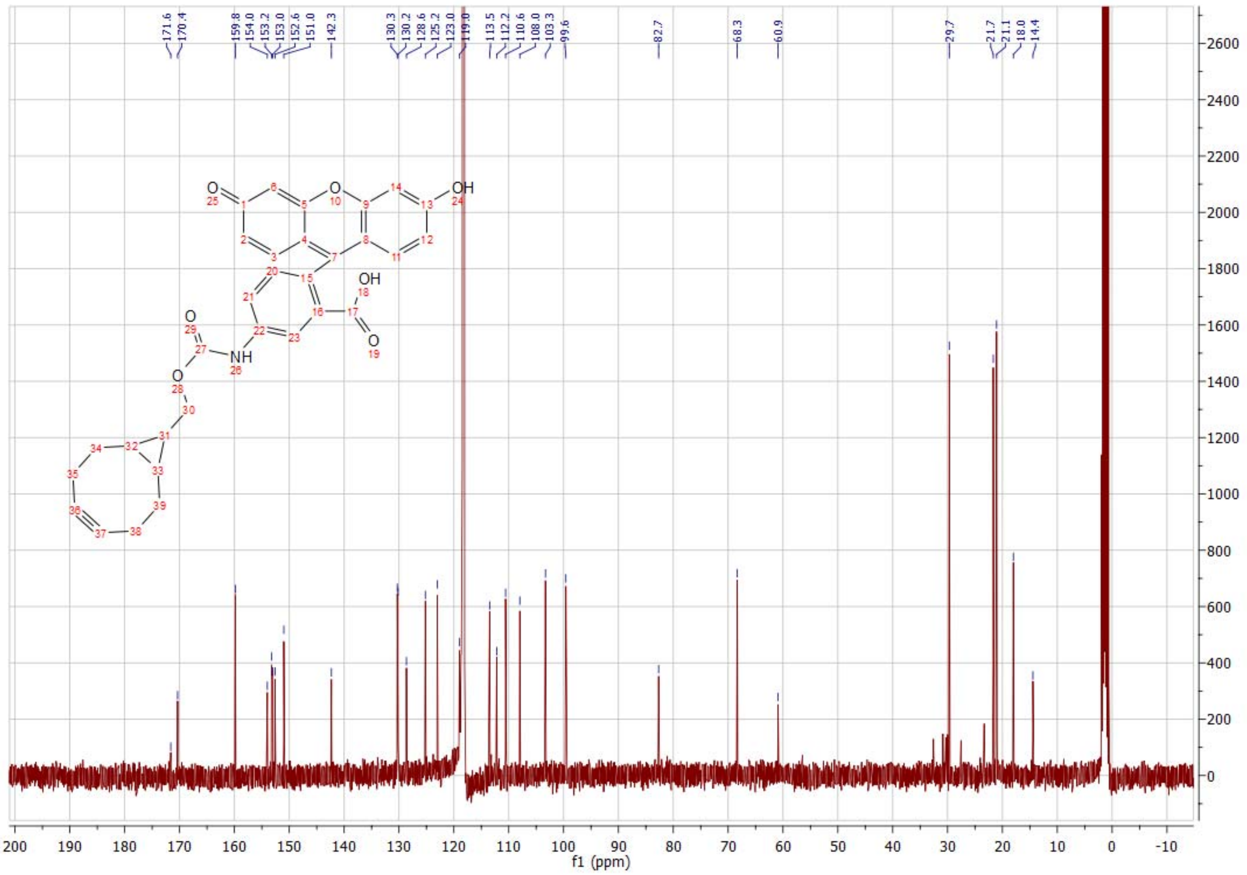

Fig S1.8. ^{13}C NMR (100 MHz, CD_3CN) of compound **2**.

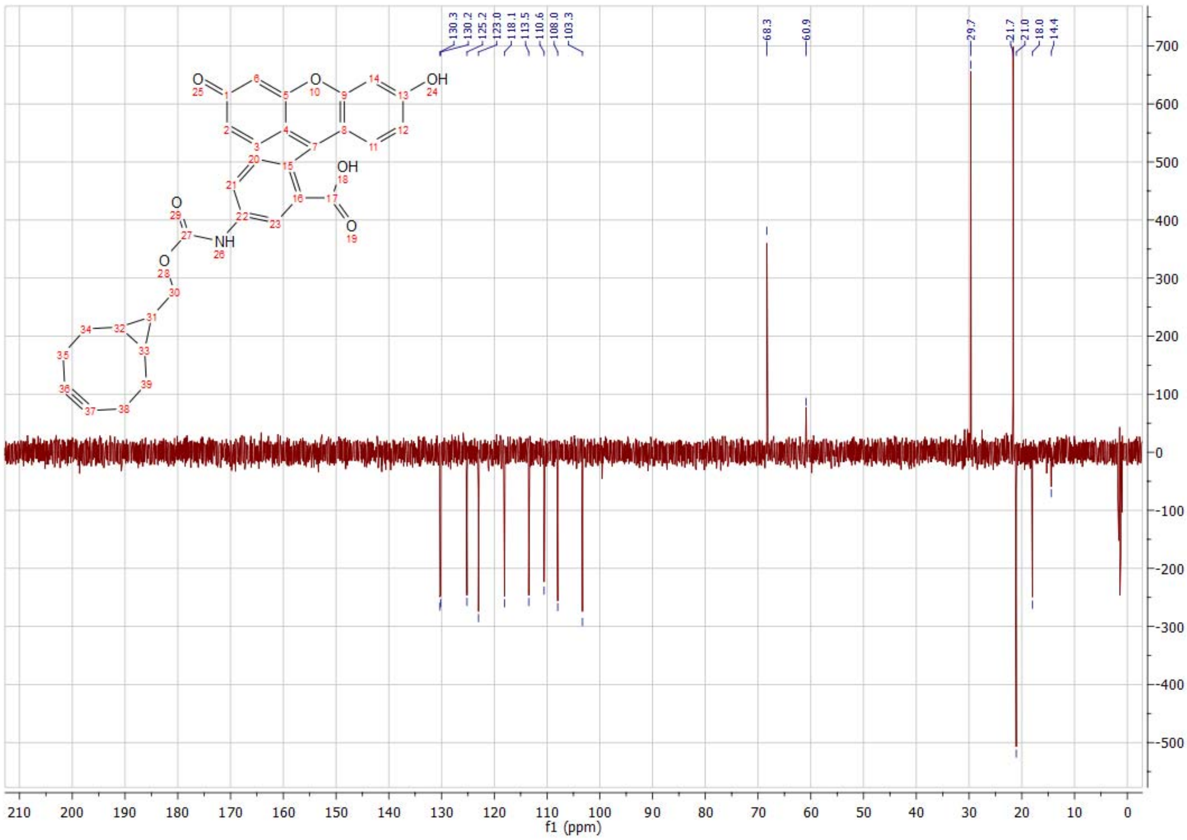

Fig S1.9. DEPT 135 (75 MHz, CD_3CN) of compound **2**.

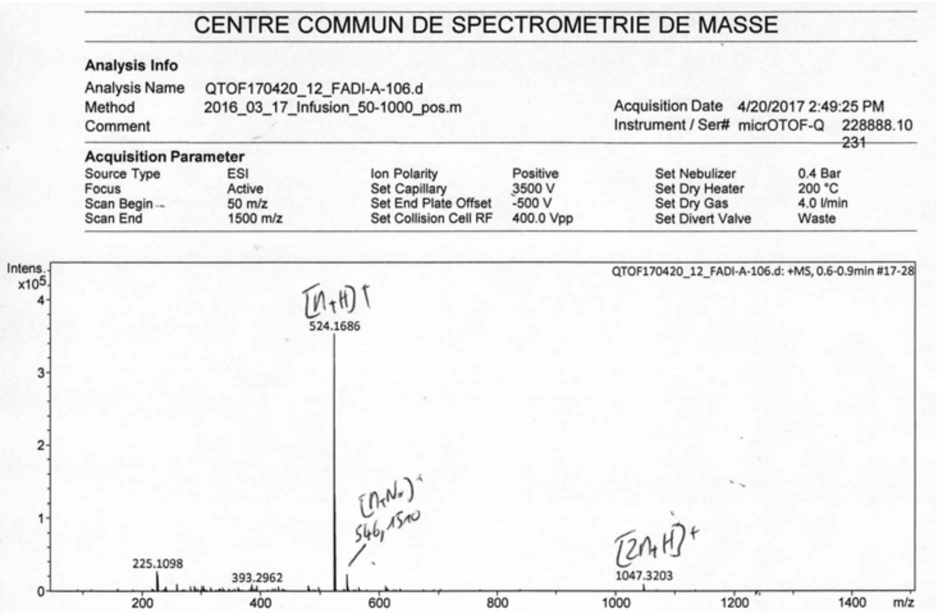

Fig S1.10. HRMS (positive ion mode) of compound **2**.

2.3 Encapsulation of BCN-Fluo inside GVs

GVs preparation, filtration, and observation was similar to what described in the main text.
In particular, BCN-Fluo-containing DOPC GVs (0.5 mM) were prepared by the natural swelling method, by including BCN-Fluo (5.7 M stock concentration in EtOH) in the I-solution (5 mM Na-bicine, pH 8.5, 200 mM sucrose), so that the final BCN-Fluo concentration is 5.7 mM and 0.1% v/v EtOH. The O-solution was similarly prepared, but without BCN-Fluo and by substituting 200 mM sucrose with 200 mM glucose. The sugars in the I- and O-solutions were introduced for comparison purposes.

2.4 Estimation of BCN-Fluo partition constant

As we have discussed elsewhere,[3] confocal images of GVs that bind a membrane-soluble fluorescent compound (and in the presence of the same free compound in inner/outer aqueous phases, see Figs S1.2-S1.3) can be analysed quantitatively in order to estimate the partition constant. The molar fraction-based partition constant K_x is defined in terms of molar fractions of the solute of interest in the lipid phase and in the aqueous phase, as it follows:

(Equation S2.1)
$$K_x = \frac{x_{membrane}}{x_{aqueous}} = \frac{\dfrac{N_m}{N_m + L}}{\dfrac{N_{aq}}{N_{aq} + W}} \approx \frac{N_m}{N_{aq}} \frac{W}{L}$$

where N_m and N_{aq} represent the number of probe molecules in the lipid phase and in the aqueous phase, respectively, whereas L and W represent the number of molecules of lipids and water, respectively (the buffer molecules are considered included in W for simplicity). The right-hand side approximation shown in Equation S2.1 holds in typical experimental conditions, i.e., $N_m << L$ and $N_{aq} << W$. The values of L and W can be conveniently calculated from lipid concentration ($W/L = 1000/18C$), where C is the lipid molar concentration; note that the density of aqueous phase is fixed to 1 g/mL). An experimental measure of N_m and N_{aq} (or of their ratio) will allow the determination of K_x.

In order to proceed, we shall simplify the system and consider all vesicles as spherical, unilamellar, and monodispersed (with radius r equal to the average GV radius). Geometrical parameters such as total intra-vesicle volume, total membrane volume, total extra-vesicle volumes can be easily

computed from lipid concentration and typical lipid molecular size (head group of lipids in lamellar phase ca. 0.7 nm^2, tail length ca. 1.8 nm).

The confocal image of a GV is the 2D projection (see Fig S2.11) of an illuminated volume V_s ($m \times n \times h$) where h is the thickness of the slice taken by confocal imaging. For a 63× objective, h is of the order of 1 μm; in our specific case, h = 0.772 μm. Note that V_s can be partitioned in three regions: $V_{in,s}$, $V_{out,s}$, and $V_{m,s}$, referring to intra-vesicle, extra-vesicle and membrane regions. If the GV radius $r >> h$, these volumes can be easily determined under a cylindrical approximation. While $V_{in,s}$ and $V_{out,s}$ are easily determined knowing r and mnh, $V_{m,s}$ is calculated by considering the bilayer thickness δ (ca. 4 nm) ($V_{m,s} \approx 2\pi rh\delta$).

Digital image analysis allows the determination of 2D integrated (sum over the pixel) fluorescence inside the vesicle, outside the vesicle and of the membrane ($\Phi_{in,s}$, $\Phi_{out,s}$, and $\Phi_{m,s}$, respectively). These values are proportional to the number of fluorescent molecules (the probe) in the corresponding illuminated volumes (which is considered as homogeneously illuminated by the laser beam). The concentrations $C_{k,s}$ of probe molecules in the illuminated volume are considered homogeneous as well, and therefore proportional to $f_{k,s} = \Phi_{k,s}/V_{k,s}$ in each region (f values actually represent 3D fluorescence densities).

The concentration of the probe in the whole sample is supposed to be equal to the concentration of the probe in the illuminated volume ($C_{k,\text{whole sample}} = C_{k,s}$), and therefore the overall number of probe molecules in the three regions (inside vesicles, outside vesicles, membrane) can be estimated as $N_k = V_k C_k \propto V_k \, \Phi_{k,s}/V_{k,s}$.

Assuming the same quantum yield of the probe in all regions (this is necessary in order to eliminate the proportional constant which links fluorescence and concentration), one finally gets Equation S2.2:

(Equation S2.2)
$$\frac{N_m}{N_{aq}} = \frac{\Phi_{m,s}\frac{V_m}{V_{m,s}}}{\Phi_{in,s}\frac{V_{in}}{V_{in,s}} + \Phi_{out,s}\frac{V_{out}}{V_{out,s}}}$$

All values in the right-hand side of Equation S2.2can be measured or computed.

K_x has been computed for a number of GVs, resulting in 1.5 ± 0.5 × 10^5 (n = 7). This corresponds to $\Delta G°_{partition}$ = –29.6 kJ/mol = –7.1 kcal/mol. Considering that the insertion of a CH_2 group in the lipid membrane corresponds, on the average, to –0.8 kcal/mol,[9-11] our estimate fits with the insertion of ca. 9 methylene groups (or equivalent moiety) in the lipid bilayer. It is therefore

reasonable to say that BCN-Fluo interacts with phospholipid membrane by inserting the hydrophobic cyclooctyne-based BCN moiety in the bilayer.

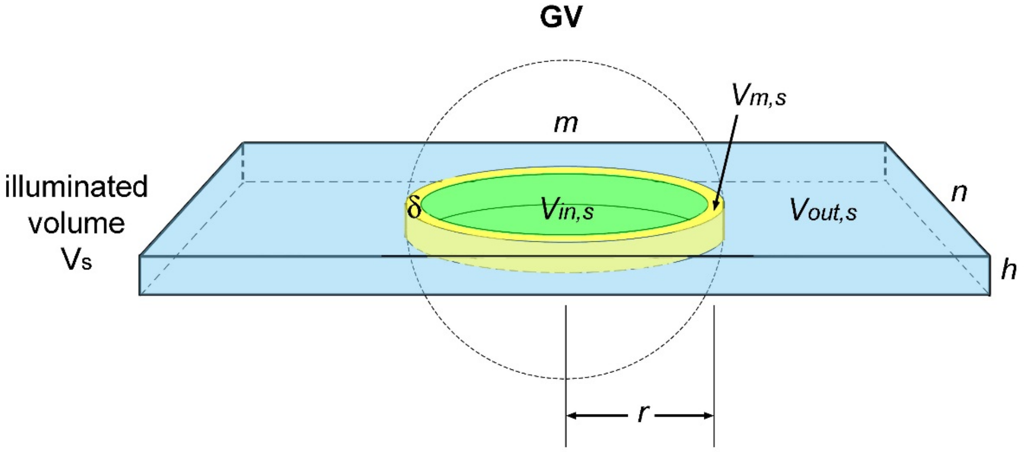

Fig S2.11. Idealized scheme of optical sectioning of a GV by confocal microscopy. The image is supposed to be focused on the GV great circle, so that the measured radius actually corresponds to the GV radius. The 2D image is a projection of the illuminated volume V_s ($m \times n \times h$) in two dimensions. The illuminated volume is sectioned in three parts: $V_{in,s}$, $V_{m,s}$, and $V_{out,s}$, as indicated. If $r >> h$, the GV curvature can be neglected and volumes can be easily computed under cylindrical approximation. While the actual membrane thickness is δ (ca. 4 nm, well below the optical resolution limit), in confocal 2D images the membrane appears in finite thickness. Its integrated fluorescence is computed from the brightness $f_{m,s}$ of the corresponding pixels. When $f_{m,s}$ is determined, however, we took into account the true membrane volume (i.e., $V_{m,s}$ is based on pixels for measuring the 2D integrated fluorescence, whereas it is based on δ for calculating the 3D fluorescence density).

3. References

[1] Baskin JM, Prescher JA, Laughlin ST, Agard NJ, Chang PV, Miller IA, et al. Copper-free click chemistry for dynamic in vivo imaging. PNAS. 2007;104: 16793–16797. doi:10.1073/pnas.0707090104

[2] Zlatopolskiy BD, Kandler R, Kobus D, Mottaghy FM, Neumaier B. Beyond azide–alkyne click reaction: easy access to 18F-labelled compounds via nitrile oxide cycloadditions. Chem Commun. 2012;48: 7134–7136. doi:10.1039/C2CC31335A

[3] Le Chevalier Isaad A, Carrara P, Stano P, Krishnakumar KS, Lafont D, Zamboulis A, et al. A hydrophobic disordered peptide spontaneously anchors a covalently bound RNA hairpin to giant lipidic vesicles. Org Biomol Chem. 2014;12: 6363–6373. doi:10.1039/c4ob00721b

[4] Kiick KL, Saxon E, Tirrell DA, Bertozzi CR. Incorporation of azides into recombinant proteins for chemoselective modification by the Staudinger ligation. PNAS. 2002;99: 19–24. doi:10.1073/pnas.012583299

[5] Saxon E, Bertozzi CR. Cell surface engineering by a modified Staudinger reaction. Science. 2000;287: 2007–2010. doi: 10.1126/science.287.5460.2007

[6] Codelli JA, Baskin JM, Agard NJ, Bertozzi CR. Second-Generation Difluorinated Cyclooctynes for Copper-Free Click Chemistry. J Am Chem Soc. 2008;130: 11486–11493. doi:10.1021/ja803086r

[7] Jewett JC, Sletten EM, Bertozzi CR. Rapid Cu-Free Click Chemistry with Readily Synthesized Biarylazacyclooctynones. J Am Chem Soc. 2010;132: 3688–3690. doi:10.1021/ja100014q

[8] de Almeida G, Sletten EM, Nakamura H, Palaniappan KK, Bertozzi CR. Thiacycloalkynes for Copper-Free Click Chemistry. Angew Chem Int Ed. 2012;51: 2443–2447. doi:10.1002/anie.201106325

[9] Peitzsch RM, McLaughlin S. Binding of acylated peptides and fatty acids to phospholipid vesicles: Pertinence to myristoylated proteins. Biochemistry. 1993;32: 10436–10443. doi:10.1021/bi00090a020

[10] Pool CT, Thompson TE. Chain length and temperature dependence of the reversible association of model acylated proteins with lipid bilayers. Biochemistry. 1998;37: 10246–10255. doi:10.1021/bi980385m

[11] Tanford C. The hydrophobic effect: formation of micelles and biological membranes. Wiley; 1980.
