# Supplementary figures and images for: Rapid purification of giant lipid vesicles by microfiltration
Source: PLoS One. 2018 Feb 16;13(2):e0192975. doi: 10.1371/journal.pone.0192975 (PMC5815610; doi:10.1371/journal.pone.0192975)

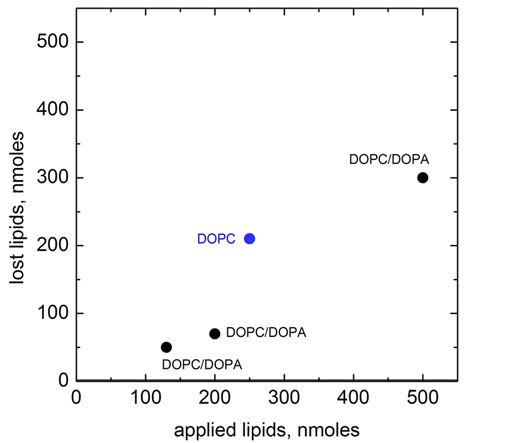

Supplement: S1 Fig — Variation of the amount of lost lipid (stacked on the filter) with the amount of applied lipids (data refer to filtrations carried out at ΔP = 400 mbar). (TIF) [file pone.0192975.s002.tif]

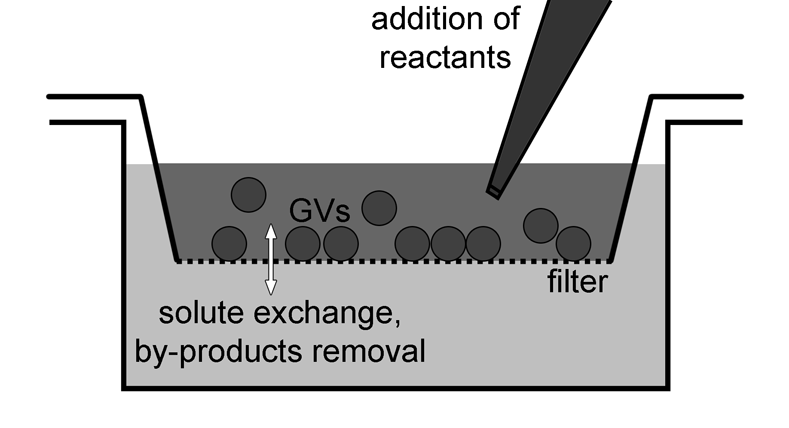

Supplement: S2 Fig — Schematic representation of a two-chamber device (sometimes referred to by the tradename Transwells®, Corning) and its hypothetical use for experiments involving GVs (intravesicle reaction, GV transformation, selection experiments, GV growth, etc.). (TIF) [file pone.0192975.s003.tif]
